# Serum apolipoprotein H determines ferroptosis resistance by modulating cellular lipid composition

**DOI:** 10.1038/s41419-024-07099-2

**Published:** 2024-10-01

**Authors:** Xiang He, Jiahui Zhang, Masha Huang, Jie Wang, Simin Yang, Xiang Yu, Yingjie Xu, Wen Yang

**Affiliations:** 1 Department of Biochemistry and Molecular Cell Biology, Shanghai Key Laboratory for Tumor Microenvironment and Inflammation, Shanghai Jiao Tong University School of Medicine, Shanghai, China; 2 Core Facility of Basic Medical Sciences, Shanghai Jiao Tong University School of Medicine, Shanghai, China

**Keywords:** Necroptosis, Lipid peroxides

## Abstract

Ferroptosis is a regulated cell death process dependent on iron, triggered by the accumulation of lipid peroxidation. The environmental context significantly impacts cellular sensitivities to ferroptosis. Serum, constituting the extracellular fluid composition in vivo, provides crucial environmental biomolecules. In this study, we investigated the influence of sera on ferroptosis induction, pinpointing the serum protein apolipoprotein H (APOH) as a pivotal inhibitor of ferroptosis. Moreover, we elucidated that APOH suppresses ferroptosis by activating the phosphoinositide 3-kinase (PI3K)-AKT-sterol regulatory element-binding proteins (SREBPs) pathway, thereby elevating stearoyl-CoA desaturase (SCD) levels and augmenting cellular monounsaturated fatty acid-containing phospholipids (MUFA-PLs). Furthermore, ApoHinfer, the peptide derivative of the active region of APOH, mimics its ferroptosis inhibitory activity. Our findings underscore the critical role of serum protein APOH in the inhibition of ferroptosis and indicates potential therapeutic applications in treating cancer and diseases associated with ferroptosis.

## Introduction

Ferroptosis is a cell death triggered by the accumulation of lipid peroxidation due to metabolic dysfunctions [1, 2]. Increasing evidence suggests that variations in the efficiency of ferroptosis induction occur at both organismal and cellular levels, highlighting the influence of the extracellular environment on ferroptosis [3, 4]. Extracellular environments play a pivotal role in determining sensitivity to ferroptosis. For example, when cells reach higher confluence, many cell types develop resistance to ferroptosis due to reduced biosynthesis of polyunsaturated fatty acid-containing phospholipids (PUFA-PLs), which is regulated by acyl-CoA synthetase long chain family member 4 (ACSL4) [5–7]. Notably, the addition of exogenous monounsaturated fatty acids (MUFAs) to the culture medium induces ferroptosis resistance by displacing oxidizable PUFAs from membrane phospholipids [8]; this highlights the crucial role of extracellular components in influencing the occurrence of ferroptosis.

Serum, similar to the extracellular fluid composition, is a crucial extracellular environments component in vivo. It provides biomolecules that regulate cellular metabolism and ferroptosis. A recent study revealed that serum cystine can be reduced to cysteine, essential for cytosolic glutathione (GSH) synthesis [9, 10]. GSH could then be used by GSH peroxidase 4 (GPX4) to convert lipid peroxides to non-toxic lipid alcohol [11]. Moreover, supplying cancer cells with albumin during treatment with an inhibitor of the mammalian target of rapamycin (mTOR) boosts albumin breakdown in lysosomes. This process releases cysteine, countering ferroptosis induced by extracellular cystine deprivation [12]. These studies underscore the impact of serum composition on ferroptosis. While ferroptosis inducers are effective in cultured cells, few interventions have proven effective in vivo [13]. This prompts speculation about the crucial regulatory role of the in vivo environment, particularly serum, in modulating ferroptosis. However, despite the importance of serum in regulating ferroptosis, understanding of its influence on cellular sensitivity to ferroptosis remains limited because of its complexity.

In this study, we aimed to systematically unveil serum components that is essential for cellular sensitivity to ferroptosis based on the diverse ferroptosis induction abilities among cells cultured in different sera. Through a process of purification and systematic identification of the active serum components, we revealed APOH, an apolipoprotein involved in lipid metabolism for lipid transport, as a potent ferroptosis inhibitor. We discovered that APOH exerts this inhibitory effect by increasing cellular levels of MUFA-PLs via the PI3K-AKT-SREBP-SCD signaling pathway, a novel function of APOH in regulating lipid composition and protecting against ferroptosis.

## Results

### Serum changes the cell sensitivity to ferroptosis

**Fig. 1:**
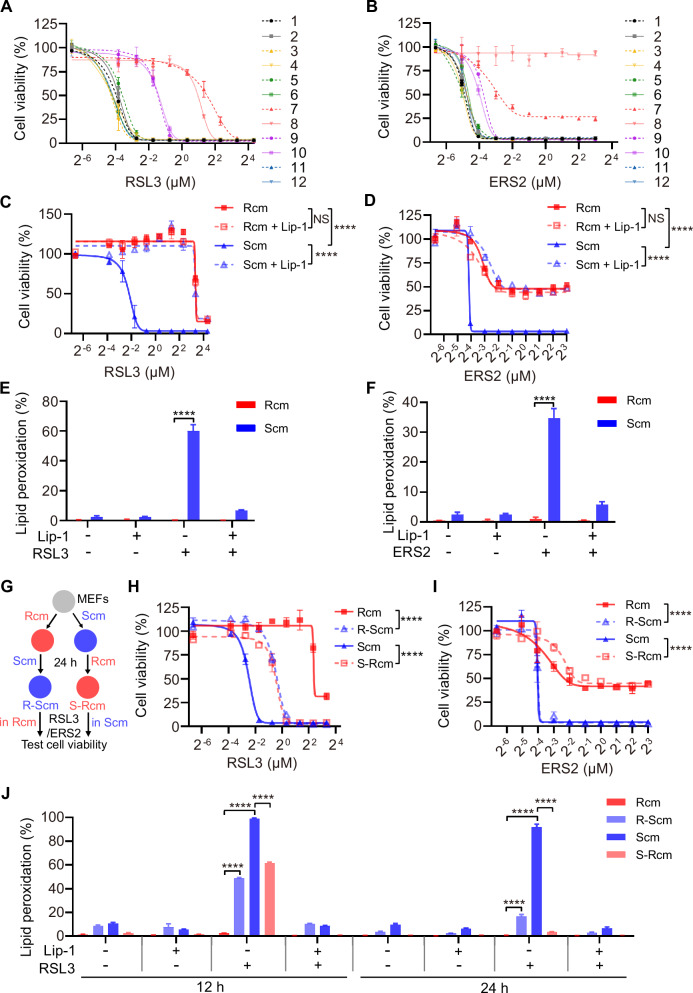
Serum changes the sensitivity of cells to ferroptosis. Cell viability of MEFs cultured with 10% FBS-1 to FBS-12 and treated with different doses of RSL3 (**A**) or ERS2 (**B**) for 24 h. Cell viability of MEFs cultured in Rcm or Scm and treated with different doses of RSL3 (**C**) or ERS2 (**D**) with or without liproxstatin-1 (Lip-1, 1 μM) for 24 h. Rcm, R-FBS complete medium; Scm, S-FBS complete medium. Lipid peroxidation level of MEFs that were treated with 2 μM RSL3 for 2 h (**E**), 2 μM ERS2 for 4 h (**F**) with or without Lip-1 (1 μM) for 2 h before staining with 2.5 μM BODIPY-C11 (C11). **G** Scheme for the procedure of MEFs originally cultured in Rcm or Scm and then switched to Scm or Rcm for 24 h, followed by treatment with RSL3/ERS2 dissolved in the original Rcm or Scm medium, and cell viability was assessed after 24 h. R-Scm, MEFs culture medium changed from Rcm to Scm; S-Rcm, MEFs cultured medium changed from Scm to Rcm. Cell viability of MEFs treated with different doses of RSL3 (**H**) or ERS2 (**I**) dissolved in original medium for 24 h according to the procedure shown in Fig. 1G. **J** Lipid peroxidation level of MEFs cultured in indicated medium for 12 h or 24 h and treated with 2 μM RSL3 with or without Lip-1(1 μM) for 2 h before staining with 2.5 μM C11. Data are presented as mean ± s.d., *n* = 3 independent repeats. Unpaired, two-tailed *t*-test; ****P < 0.0001. NS not significant.

Ferroptosis is susceptible to influences from the extracellular environment. Owing to the complex composition and variations in the sera of different individuals, and the crucial role played by serum in this environment, it was hypothesized that different sera exhibit varying abilities to induce ferroptosis. To test this hypothesis, mouse embryonic fibroblasts (MEFs) were cultured with fetal bovine serum (FBS) that was available on the market at that time, sourced from different countries and companies. The ferroptosis-inducing abilities of these sera were systematically evaluated under these conditions. Notably, MEFs cultured in different sera showed varied responses to ferroptosis induction by RSL3/ERS2 (Fig. 1A, B). The MEFs cultured in FBS-7 and 8 exhibited the highest resistance to ferroptosis; however, those cultured in FBS-1 and 2 displayed higher sensitivity (Fig. 1A, B). FBS-1 was designated as S-FBS (FBS sensitive to ferroptosis) because it sensitizes MEFs to ferroptosis. Complete medium (cm) containing 10% S-FBS is referred to as 10% Scm or Scm. FBS-7 was named R-FBS (FBS resistant to ferroptosis) because it confers resistance to ferroptosis in MEFs. Complete medium with 10% R-FBS is referred to as 10% Rcm or Rcm.

Ferroptosis and lipid peroxidation of MEFs cultured in Scm could be rescued by ferroptosis inhibitor liproxstatin-1 (Lip-1), which inhibits lipid peroxidation; the ferroptosis-inhibiting activity of Rcm is comparable to that of Lip-1 (Fig. 1C–F). Additionally, it was observed that Rcm conferred resistance to ferroptosis induction in all cell lines tested, including Hepa 1-6, H1299, BT549, C2C12, CT26, 4T1, and SK-OV-3; conversely, cells cultured in Scm were sensitive to ferroptosis induction, consistent with the effects observed in MEFs (Fig. S1A–G).

To further elucidate the effect of serum on the ability to induce ferroptosis, the culture medium in MEFs was exchanged by swapping from Rcm to Scm for 24 h (R-Scm) and ferroptosis was induced by adding RSL3/ERS2 to Rcm, and vice versa (Fig. 1G). It was observed that MEFs cultured in R-Scm exhibited greater susceptibility to ferroptosis than those cultured in Rcm, despite the dissolution of RSL3/ERS2 in Rcm. Similarly, compared with cells cultured in the Scm, MEFs showed significantly increased resistance to ferroptosis after the medium was changed to Rcm for 24 h (S-Rcm), despite the dissolution of RSL3/ERS2 in Scm (Fig. 1H, I). These results indicate that the pharmacological activity of the ferroptosis inducers RSL3/ERS2 is independent of the type of culture medium in which they are dissolved. Moreover, the effect of serum on lipid peroxidation generation was consistent with ferroptosis induction (Fig. 1J). These results suggest that serum composition affects the sensitivity of cells to ferroptosis rather than influencing the pharmacological activity of ferroptosis-inducing agents.

### Serum protein APOH inhibits ferroptosis

**Fig. 2:**
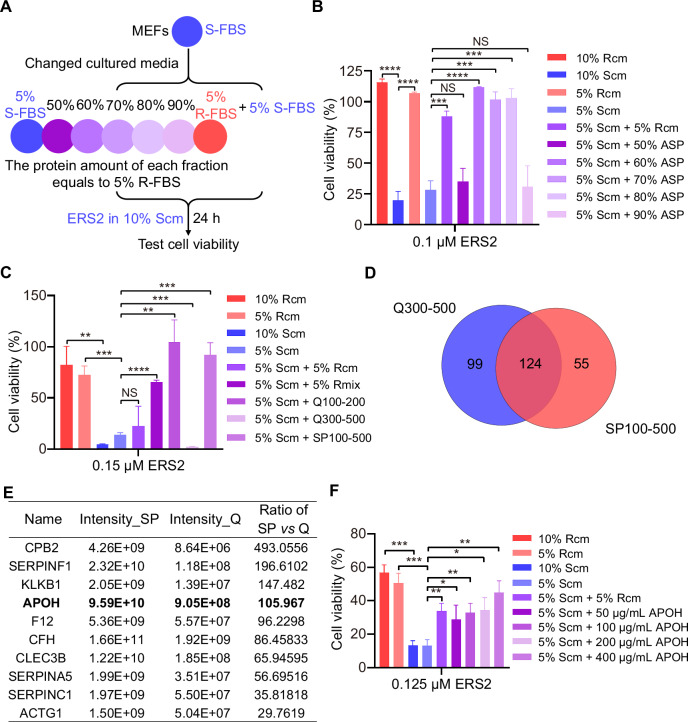
Serum protein APOH inhibits ferroptosis. **A** Scheme for the procedure of MEFs cultured in Scm changed to medium containing 5% S-FBS adding 5% S-FBS or 5% R-FBS or 50–90% ASP or not for 24 h. The protein amount of 50–90% ASP was equal to 5% R-FBS. MEFs were treated with ERS2 in Scm for 24 h before cell viability was tested with CCK-8. ASP, ammonium sulfate precipitation. **B** Cell viability of MEFs cultured in indicated medium for 24 h and treated with ERS2 (0.1 μM) for 24 h in Scm according to the procedure shown in (**A**). **C** Cell viability of MEFs cultured in 5% or 10% of Rcm or Scm, or medium containing 5% S-FBS with the addition of various components, including 5% R-FBS, 5% Rmix, Q100–200, Q300–500, or SP100–500 for 24 h. Subsequently, the cells were treated with ERS2 (0.15 μM) for an additional 24 h in Scm. The protein amount of Rmix, Q100–200, Q300–500, and SP100–500 equals 5% R-FBS. Q100–200 or Q300–500, protein eluted with 100–200 mM or 300–500 mM NaCl in HiTrap-Q (Q) column; SP100–500, protein eluted with 100–500 mM NaCl in HiTrap-SP (SP) column. Rmix, the mixture of 60–80% ASP of R-FBS. **D** Venn diagram illustrates the overlapping proteins detected in the SP100–500 and Q300–500 fractions identified by mass spectrometry (MS). **E** Protein list of overlap proteins of SP100-500 and Q300–500 identified by MS and ranged by the intensity ratio of SP100–500 to Q300–500. **F** Cell viability of MEFs cultured in Scm changed for medium containing 5% S-FBS adding 5% R-FBS or different amounts of purified APOH for 24 h and treated with ERS2 (0.125 μM) for 24 h in Scm. Data are presented as mean ± s.d., *n* = 3 independent repeats. Unpaired, two-tailed *t*-test; *P < 0.05, **P < 0.01, ***P < 0.001, ****P < 0.0001. NS not significant.

Some proteins present in R-FBS could responsible for the increase of cellular resistance to ferroptosis. To test this hypothesis, ammonium sulfate precipitation (ASP) was used to fractionate various proteins from R-FBS. The principle of ASP involves precipitating proteins by competing for water molecules, which reduces the hydration layer around proteins and leads to aggregation and precipitation [14]. Different proteins precipitate at specific concentrations of ammonium sulfate. Using this characteristic of ammonium sulfate, we can separate the proteins in R-FBS and verify their ferroptosis resistance activity. Each fraction of R-FBS was added to S-FBS and examined to evaluate whether the addition enhanced the resistance of cells cultured in Scm to ferroptosis (Figs. 2A and 2A). The effect of ASP fractions ranging from 50% to 90% in R-FBS was investigated, with a 10% increase per fraction, on cellular ferroptosis. However, due to insufficient protein amounts, the 30% and 40% fractions were not further investigated (Fig. S2B). The culture medium of MEFs was changed from Scm to complete medium containing 5% S-FBS (v/v) and each ASP fraction with a protein amount equal to that of 5% S-FBS (5% Scm + each ASP fraction) (Fig. 2A). It was observed that the 60%, 70%, and 80% ASP fractions induced resistance to ferroptosis; conversely, the 50% and 90% fractions did not show this effect (Figs. 2B and S2C). Consequently, the mixture of 60–80% ASP fraction (Rmix) inhibited ferroptosis more significantly than R-FBS, suggesting that Rmix enriched the active components (Fig. S2D). The active proteins of Rmix were fractionated using ion-exchange column chromatography. Rmix was separately loaded onto anion-exchange HiTrap-Q (Q) and cation-exchange HiTrap-SP (SP) chromatography columns (Fig. S2A). The fractions eluted with 100–200 mM NaCl in Q (Q100–200) and 100–500 mM NaCl in SP (SP100–500) retained their ferroptosis-inhibiting activity; however, those eluted with 300–500 mM NaCl in Q (Q300–500) did not (Figs. 2C and S2E).

The fractions were then analyzed using sodium dodecyl sulfate-polyacrylamide gel electrophoresis (SDS-PAGE) and Coomassie brilliant blue staining. It was discovered that the band with a molecular weight between 50 kDa and 70 kDa was enriched in the active fractions (Q100–200 and SP100–500), but Q300–500 was not (Fig. S2F). SP100–500 and Q300–500 mass spectrometry (MS) analyses were performed (Fig. 2D, E, and Table S1). Because the two fractions were originally from Rmix but had different activities, it was speculated that the ferroptosis-inhibiting protein should be present in both fractions but enriched in the SP100–500 fraction. Among all the candidate proteins selected using this approach, APOH was the only protein with functions related to lipid metabolism [15], and its molecular size (55 kDa) falls within the 50 kDa to 70 kDa range (Fig. 2E).

To determine the ferroptosis-inhibiting effect of APOH, recombinant mouse APOH tagged with His was purified and added to 5% Scm at different concentrations (Fig. S2G); APOH inhibited lipid peroxidation (Fig. S2H) and ferroptosis dose-dependently (Fig. 2F). Notably, the protein levels of APOH were significantly higher in R-FBS (FBS-7) and FBS-8, which were more resistant to ferroptosis than the other sera (Figs. S2I and 1A, B). In addition, it was observed that the APOH protein level in R-FBS was ~15 times higher than that in S-FBS (FBS-1) (Fig. S2J). These results suggested that APOH plays an essential role in protecting MEFs from ferroptosis.

### APOH increases the fatty acid and cholesterol biosynthesis pathway

**Fig. 3:**
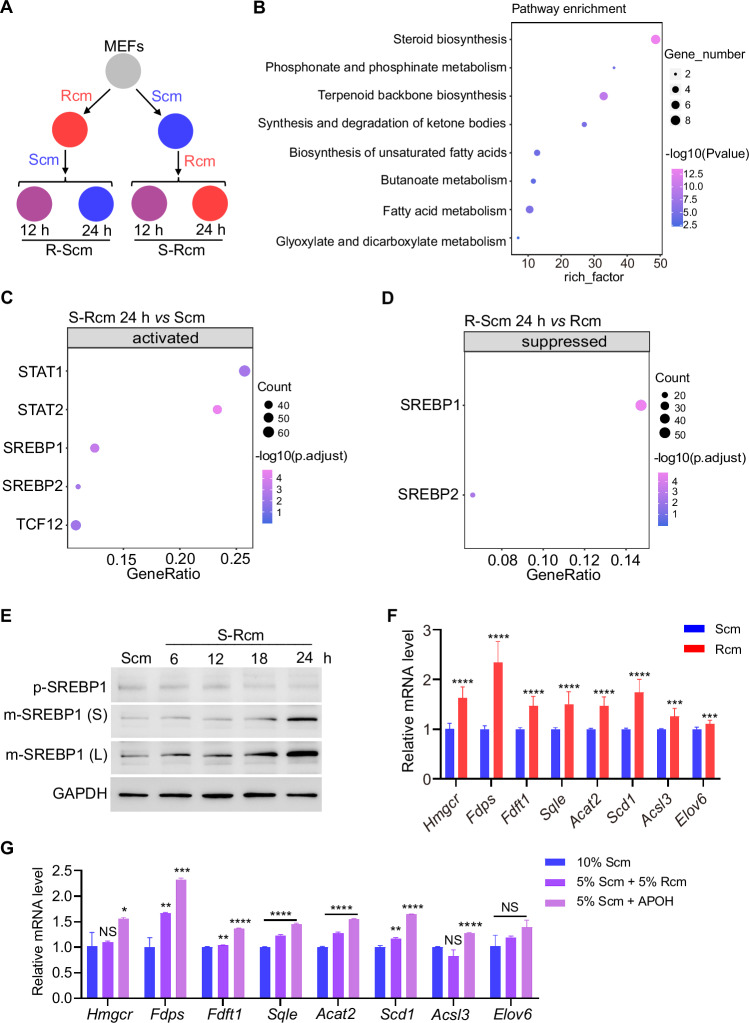
APOH increased the fatty acid and cholesterol biosynthesis pathway. **A** Scheme for the procedure of MEFs’ original cultured medium that was exchanged for 12 h or 24 h or remain unchanged. R-Scm 12 h, R-Scm 24 h, MEFs culture medium changed from Rcm to Scm for 12 h or 24 h; S-Rcm 12 h, S-Rcm 24 h, MEFs cultured medium changed from Scm to Rcm for 12 h or 24 h. **B** KEGG pathway enrichment analysis for differentially expressed genes (DEGs) altered by the culture medium exchanged. **C**, **D** Transcription factor analysis of DEGs in MEFs after changing the medium. The transcription factors of MEFs activated by changing the medium from Scm to Rcm for 24 h (**C**) and suppression by changing the medium from Rcm to Scm for 24 h (**D**). **E** Western blot analysis of the protein levels of SREBP1 in MEFs cultured in Scm and in cells in which the medium was changed from Scm to Rcm for the indicated time. p-SREBP1, precursor SREBP1. m-SREBP1, mature SREBP1. S, short exposure; L, long exposure. **F** the mRNA level of the indicated SREBP1 target genes of MEFs cultured in Scm or Rcm by RT-qPCR. **G** mRNA level of the indicated SREBP1 target genes of MEFs cultured in Scm changed medium to 5% Scm + 5% Rcm (containing 5% S-FBS and 5% R-FBS) or 5% Scm + APOH (containing 5% S-FBS and 120 μg/mL APOH) for 12 h by RT-qPCR. Data are presented as mean ± s.d., *n* = 3 independent repeats. Unpaired, two-tailed *t*-test; *P < 0.05, **P < 0.01, ***P < 0.001, ****P < 0.0001. NS not significant.

Given the effect of serum on cellular sensitivity to ferroptosis, this study aimed to elucidate the mechanisms by which R-FBS/APOH mediates cellular resistance to ferroptosis. The transcriptomes of MEFs cultured in Rcm, Scm, and exchange medium for 12 h (referred to as R-Scm 12 h or S-Rcm 12 h, respectively) and 24 h (referred to as R-Scm 24 h or S-Rcm 24 h, respectively) was analyzed using RNA sequencing (RNA-seq) (Fig. 3A). Differentially expressed genes (DEGs) whose expression levels were altered by changes in the culture medium were analyzed. DEGs were selected based on a cutoff fold-change of 1.5. Kyoto Encyclopedia of Genes and Genomes (KEGG) pathway analysis revealed that fatty acid and cholesterol biosynthesis pathways were enriched in these DEGs (Figs. 3B and S3A, B). The activity of the transcription factor SREBP1 was activated when switching from Scm to Rcm; however, it was inhibited when switching from Rcm to Scm (Fig. 3C, D). SREBP1 is a master transcription factor that regulates the synthesis of fatty acids and cholesterol. The mature form of SREBP1 (mSREBP1) is translocated to the nucleus to regulate its downstream transcriptional targets [16]. It was discovered that the protein level of mSREBP1 is increased upon changing the medium from Scm to Rcm (Fig. 3E).

Genes related to fatty acid and cholesterol biosynthesis in MEFs were confirmed by quantitative PCR (qPCR). The expression of these genes, such as *Scd1*, was consistently higher in cells cultured in Rcm than in those cultured in Scm (Figs. 3F and S3C) and was upregulated in MEFs upon switching from Scm to Rcm (Fig. S3D). To validate whether APOH alters the expression of these genes in MEFs cultured in Scm, the gene expression levels between MEFs cultured in 5% Scm and 5% Scm + APOH (complete medium containing 5% S-FBS supplemented with 120 μg/mL APOH) was compared using qPCR. The results showed that APOH increased the expression of genes associated with fatty acid and cholesterol biosynthesis (Figs. 3G and S3E).

### APOH increases the MUFA-PLs to inhibit ferroptosis

**Fig. 4:**
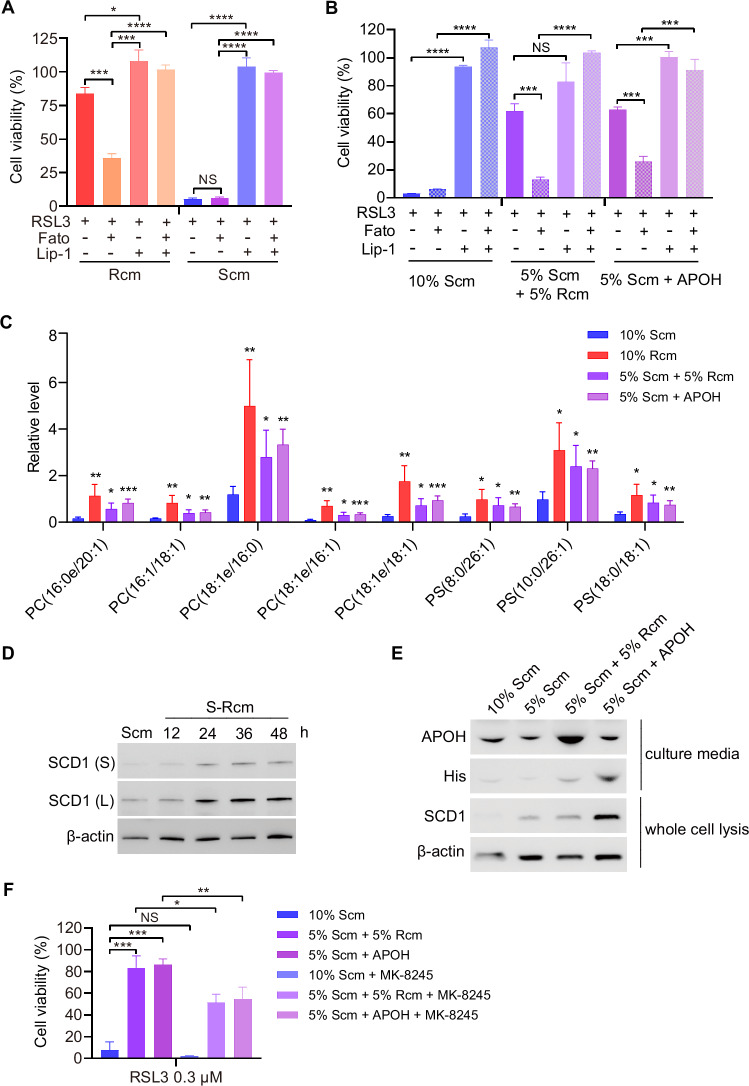
APOH increases the MUFA-PLs/MUFA-ePLs to inhibit Ferroptosis. **A** Cell viability of MEFs cultured in Scm and then changed medium to Rcm or not, treated with or without Fatostatin A (Fato, 5 μM) for 12 h before treatment with RSL3 (0.15 μM) and/or Lip-1 (1 μM) for 24 h. **B** Cell viability of MEFs cultured in Scm and then changed medium to 5% Scm + 5% Rcm, 5% Scm + APOH (120 μg/mL) or not, treated with or without Fato (5 μM) for 12 h before treatment with RSL3 (0.225 μM) and/or Lip-1 (1 μM) for 24 h. **C** Relative levels of differentially altered MUFA-PLs/MUFA-ePLs of MEFs cultured in Scm, Rcm, 5% Scm + 5% Rcm, and 5% Scm + APOH (120 μg/mL). MUFA-PLs were normalized to the corresponding mean value. *n* = 4 independent repeats. **D**, **E** Western blot analysis of the protein levels of SCD1 in MEFs cultured in Scm and in cells in which the medium was changed from Scm to Rcm for the indicated time (**D**) or to 5% Scm + 5% Rcm or 5% Scm + APOH (120 μg/mL) (**E**). (**F**) Cell viability of MEFs cultured in Scm changed medium to 5% Scm + 5% Rcm or 5% Scm + APOH (120 μg/mL) treated with or without MK-8245 (80 μM) for 24 h before treatment with RSL3 (0.3 μM). Western blot is representative of three biological replicates. Data are presented as mean ± s.d., *n* = 3 independent repeats (**A**, **B**). *n* = 4 independent repeats (**C**). Unpaired, two-tailed *t*-test; *P < 0.05, **P < 0.01, ***P < 0.001, ****P < 0.0001. NS not significant.

To explore the influence of the upregulated fatty acid and cholesterol biosynthesis pathways on ferroptosis resistance induced by Rcm/APOH, Fatostatin A (Fato), an inhibitor of SREBP [17], was utilized to suppress the biosynthesis of fatty acids and cholesterol in MEFs. Results demonstrated that the increased resistance of MEFs to ferroptosis upon changing the culture medium from Scm to Rcm or 5% Scm + APOH was mitigated by Fato (Fig. 4A, B).

Since SREBP1 is a key transcription factor in the de novo synthesis of lipids, whether R-FBS/APOH affected the lipid composition of cells was evaluated. The lipidomics of MEFs cultured in Scm, Rcm, 5% Scm + 5% Rcm (the complete medium containing 5% S-FBS and 5% R-FBS), and 5% Scm + APOH was compared; 84 different lipids were identified in the four groups (Fig. S4A). Among these lipids, 13 phospholipids (PLs) and ether PLs (ePLs, a special class of PLs) containing MUFAs were identified (Fig. S4B); the levels of 12 out of the 13 types of the PLs/ePLs were significantly higher in the Rcm than in the Scm (Fig. S4B). Additionally, an increase in the levels of 8 types of MUFA-PLs and MUFA-ePLs (ePLs containing MUFAs) in cells cultured in 5% Scm + APOH medium compared to those in cells cultured in Scm was observed (Fig. 4C). In conjunction with the gene expression data, these findings suggest that Rcm/APOH significantly alters lipid metabolism and composition. Notably, it has been reported that MUFA-PLs can confer cellular protection against ferroptosis by displacing PUFA-PLs from the plasma membrane, reducing their susceptibility to oxidation [8]. Therefore, it was hypothesized that the observed changes in lipid composition resulting from Rcm/APOH treatment might contribute to its inhibitory effect on ferroptosis.

SCD1 is a transcriptional target of SREBP1 and functions as a rate-limiting enzyme in MUFAs biosynthesis. It was observed that SCD1 protein levels increased when the medium was switched from Scm to Rcm (Fig. 4D). Consistently, the SCD1 protein level in MEFs cultured in 5% Scm + APOH was higher than that in MEFs cultured in Scm (Fig. 4E). To verify whether R-FBS/APOH inhibited ferroptosis by modulating the enzymatic activity of SCD1 in the biosynthesis of MUFAs, SCD1 inhibitor MK-8245 [18] was used for validation. Results demonstrated that the increased resistance of MEFs to ferroptosis upon changing the culture medium from Scm to 5% Scm + 5% Rcm or 5% Scm + APOH can be inhibited by MK-8245 (Fig. 4F). Therefore, R-FBS/APOH increased the cellular content of MUFA-PLs (including MUFA-PLs and MUFA-ePLs), consequently inhibiting ferroptosis.

GPX4 is a key regulator of ferroptosis [11, 19]. As a selenoprotein, the biosynthesis of GPX4 is dependent on isopentenyl pyrophosphate (IPP), an intermediate metabolite in the cholesterol biosynthesis pathway [20, 21]. Given that most genes of the cholesterol biosynthesis pathway were upregulated during the exchange from Scm to Rcm and downregulated vice versa (Fig. S3B), it was presumed that the protein biosynthesis of GPX4 plays a role in the inhibition of ferroptosis induced by R-FBS. Higher protein levels of GPX4 were found in MEFs cultured in Rcm than in those cultured in Scm (Fig. S5A), with an increase upon changing the medium from Scm to Rcm (Fig. S5B). Moreover, it was observed that the enhanced resistance of MEFs to ferroptosis induced by switching the medium from Scm to Rcm was counteracted by the addition of lovastatin (Lova) [22], an inhibitor of 3-hydroxy-3-methylglutaryl-CoA reductase (HMGCR), the rate-limiting enzyme in the cholesterol biosynthesis pathway (Fig. S5C). Furthermore, the effect of Lova on lipid peroxidation generation was consistent with ferroptosis induction (Fig. S5D). Therefore, it was suspected that R-FBS enhanced the activity of the cellular cholesterol synthesis pathway, leading to increased production of intermediate metabolites and a subsequent increase in the synthesis of GPX4. However, the increased protein levels of GPX4 upon changing from Scm to Rcm were not altered by treatment with Lova (Fig. S5B). This suggests that R-FBS increases the protein level of GPX4 independently of the cholesterol biosynthesis pathway. More importantly, it was observed that *Gpx4*-knockout MEFs cultured in Scm were sensitive to ferroptosis; in contrast, those cultured in Rcm were still resistant (Fig. S5E–G). Therefore, R-FBS inhibits ferroptosis independent of GPX4 activity.

### APOH inhibits ferroptosis by activating the AKT-SREBP1-SCD pathway

**Fig. 5:**
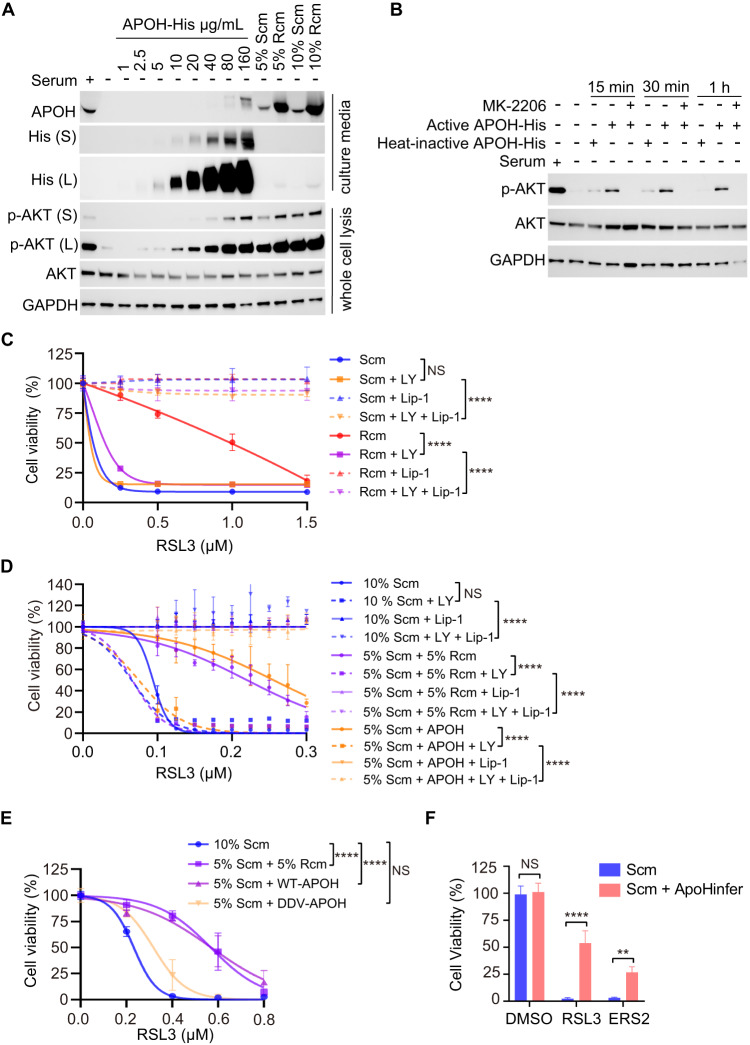
APOH inhibits ferroptosis by activating the AKT-SREBP1 pathway. **A** APOH induced the phosphorylation of AKT (p-AKT) in MEFs. Western blot analysis was performed to measure the p-AKT, AKT, and APOH. MEFs cultured in Scm were serum-starved for 12 h and then treated with the indicated concentration of APOH, or 5% or 10% of Rcm or Scm for 30 min. **B** PI3K/AKT inhibitor MK-2206 prevented the p-AKT induced by APOH. MEFs cultured in Scm were serum-starved for 12 h and then treated with 10 μg/mL APOH or heat-inactivated APOH for the indicated time with or without MK-2206 (5 μM). Western blot analysis was performed to measure p-AKT and AKT. **C** Cell viability of MEFs cultured in Scm changed medium to Rcm or not, treated with or without LY294002 (LY, 12 μM) for 12 h before treatment with different doses of RSL3 and/or Lip-1 (1 μM) for 24 h. **D** Cell viability of MEFs cultured in Scm and then changed medium to 5% Scm + 5% Rcm, 5% Scm + APOH (120 μg/mL) or not, treated with or without LY (12 μM) for 12 h before treatment with different doses of RSL3 and/or Lip-1 (1 μM) for 24 h. **E** Cell viability of MEFs cultured in Scm changed medium to 5% Scm + 5% Rcm, 5% Scm + WT-APOH (120 μg/mL), 5% Scm + DDV-APOH (120 μg/mL) or not, treated with different doses of RSL3 for 24 h. **F** Cell viability of MEFs cultured in Scm changed medium to Scm + ApoHinfer (Scm adding 300 μg/mL ApoHinfer) or not for 24 h, before treated with or without 0.4 μM RSL3 or 0.2 μM ERS2 for 24 h. Western blot is representative of three biological replicates (**A**, **B**). Data are presented as mean ± s.d., *n* = 3 independent repeats. Unpaired, two-tailed *t*-test; **P < 0.01, ****P < 0.0001. NS not significant.

SREBPs are regulated by the PI3K-AKT pathway, which serves as a molecular hub linking extracellular and intracellular stimuli to various cellular processes [23–25]. The activation of the PI3K-AKT pathway by Rcm was investigated to evaluate its effect on SREBP1. In serum-depleted MEFs cells, the activity of AKT indicated by phosphorylated AKT (p-AKT) is at a low level. It was discovered that Rcm and purified APOH significantly increased p-AKT compared to Scm (Fig. 5A). Furthermore, APOH increased p-AKT in a dose-dependent manner (Fig. 5A). The ability of APOH to activate AKT was inhibited by the PI3K/AKT inhibitor MK-2206 [26]; and heat-treated APOH was unable to activate AKT (Fig. 5B). Consistent with the downstream regulation of SREBP1, the increase in SCD1 protein levels resulting from the change in the cell culture medium from Scm to Rcm or APOH was mitigated by the AKT inhibitor, LY294002 (LY) [27], confirming the anticipated involvement of AKT in this regulatory pathway (Fig. S6A, B). Furthermore, the increased resistance of MEFs to ferroptosis observed upon changing the culture medium from Scm to 5% Scm + 5% Rcm or 5% Scm + APOH was mitigated by LY (Fig. 5C, D).

APOH binds preferentially to negatively charged phospholipids that contain five domains. The fifth domain (domain V, the domain close to C-terminal) of APOH is the most functional for the binding to anionic phospholipid [28]. The domain V (244–326, mature peptide amino acid numbering) contains two active regions. The highly positively charged region between amino acids (AA) 281–288 (CKNKEKKC) is conserved and critical for phospholipid binding [29]. The region of AA 311–317 (SSLAFWK) plays a crucial role in its insertion into lipid monolayers and the insertion ability of APOH is stronger when a higher content of negatively charged lipids is present in the membrane [30], however, its biological functions remain unclear.

It was suspected that APOH may facilitate the binding between APOH and the cell membrane, which is important for the activation of AKT. To test this hypothesis, a deleted domain V mutant variant of APOH (DDV-APOH) was generated. In contrast to wild-type APOH (WT-APOH), DDV-APOH showed reduced efficacy in augmenting p-AKT (Fig. S6C). Notably, DDV-APOH demonstrated decreased ferroptosis inhibitory activity compared to WT-APOH, providing consistent evidence of its diminished functional impact on ferroptosis inhibition (Fig. 5E). To further evaluate the efficacy of domain V of APOH in inhibiting ferroptosis, we synthesized a peptide named ApoHinfer, which combines two active regions of domain V (AA 281–288, AA 311–317) with a linker composed of two glycine residues. Consistently, MEFs cultured in Scm with ApoHinfer effectively inhibited ferroptosis induced by RSL3 and ERS2 (Fig. 5F). Additionally, it increased SCD level, similar to APOH, thereby inhibiting ferroptosis (Fig. S6D).

### APOH expression is associated with sorafenib response in patients with liver cancer

APOH is synthesized primarily in the liver and excreted into the bloodstream [15]. Various diseases and injuries can impact the expression of *APOH* mRNA in the liver [31, 32]. We suspect that the differential levels of APOH protein in serum and its regulatory function in ferroptosis might play a significant role in the occurrence and progression of liver-related diseases. Hepatocellular carcinoma (HCC), the most common type of liver cancer, is treated with sorafenib, a multi-kinase inhibitor and the only approved systemic agent for HCC [33]. Sorafenib induces ferroptosis by targeting SLC7A11 [34, 35], which imports cystine for GSH biosynthesis, and by inhibiting SCD expression [36]. Therefore, we hypothesize that APOH may interact with the therapeutic effect of sorafenib on HCC.

**Fig. 6:**
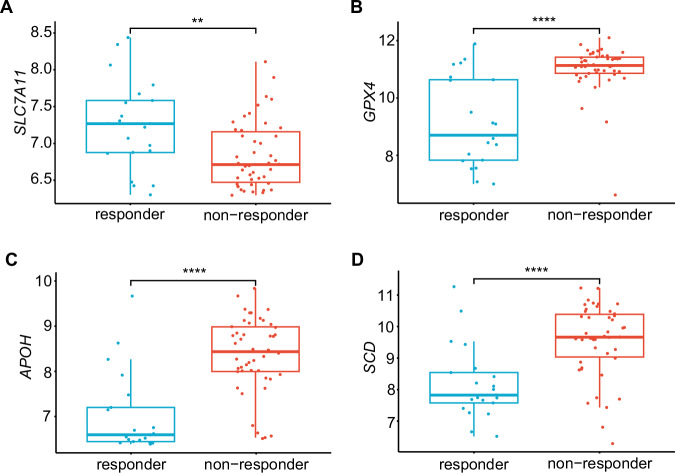
APOH expression associates with sorafenib response in patients with liver cancer. Expression of *SLC7A11* (**A**), *GPX4* (**B**), *APOH* (**C**), and *SCD* (**D**) were evaluated in patients with HCC hepatocellular carcinoma, they were categorized as 21 responders and 46 non-responders to sorafenib treatment using the GEO dataset (GSE109211). The statistical difference of the two groups was compared through the *t*-test. **P < 0.01, ****P < 0.0001.

To test this hypothesis, the GSE109211 dataset [37] from the Gene Expression Omnibus (GEO) database, which included gene expression data from 67 patients with HCC who underwent sorafenib treatment, was analyzed. Among these patients, 21 individuals were classified as sorafenib treatment responders and 46 as non-responders; conducted analysis revealed that patients with high *SLC7A11* expression responded better to sorafenib treatment (Fig. 6A). Furthermore, higher expression levels of *GPX4* were correlated with resistance to sorafenib treatment (Fig. 6B), further confirming that ferroptosis plays an important role in sorafenib treatment in this dataset [34, 36].

The conducted analysis revealed that the patients who exhibited resistance to sorafenib treatment (non-responder) had higher *APOH* levels than those who responded to sorafenib treatment (Fig. 6C). This indicates that sorafenib efficacy is associated with *APOH* levels in HCC. Additionally, the analysis revealed that patients with HCC who were resistant to sorafenib treatment exhibited relatively higher expression levels of fatty acid and cholesterol-related genes, including *SCD, SREBP1,* and *HMGCR* (Figs. 6D and S7A, B), which was highly correlated with the findings.

## Discussion

Serum provides various components to cells and serves as an important culture medium. Previous studies revealed that serum components such as transferrin [38], glutamine [38, 39], and cystine [40] modulate ferroptosis by affecting cellular redox capacity and controlling the occurrence of ferroptosis. But despite the fact that ferroptosis is a regulated cell death, none of these components involved in cellular response to extracellular signals. Our study represents the first demonstration that serum protein can directly modulate cellular lipid metabolism via signaling pathways and impacting cell sensitivity to ferroptosis. Specifically, we revealed that APOH is a pivotal protein in the serum that can confer cellular resistance to ferroptosis by modulating cellular lipid composition. Mechanistically, APOH exerts its inhibitory effect on ferroptosis by activating SREBP1, increasing SCD1 levels, and subsequently enhancing the content of MUFA-PLs/MUFA-ePLs. This study underscores the significance of serum as an extracellular milieu in regulating lipid metabolism linked to ferroptosis.

PI3K-AKT pathway act as molecular hubs that respond to extracellular signals and regulate various cellular processes [23, 24]. Our study shows that the serum protein APOH activates the PI3K/AKT-SREBP1-SCD1 pathway to increase the level of MUFA-PLs/MUFA-ePLs. The attenuation of APOH resistance to ferroptosis by SCD1 suppression (Fig. 4F), likely due to the decrease in MUFA-PLs/MUFA-ePLs, thereby increases cellular sensitivity to ferroptosis induction [8, 41]. Therefore, this study established APOH as a novel regulator of the PI3K-AKT pathway and that increasing MUFA-PLs/MUFA-ePLs levels through this regulation contributes to cell resistance to ferroptosis.

Apolipoproteins bind to lipids to form lipoproteins, primarily responsible for lipid transport [42], it can also bind to plasma membrane lipid [43]. APOH preferentially binds to negatively charged phospholipids, such as cardiolipin, phosphatidylserine (PS), and phosphatidylinositol (PI) [44, 45]. The domain V of APOH (244-326, mature protein amino acid numbering) is sufficient for binding to PI 4,5-bisphosphate (PI(4,5)P_2_) and PI 3,4,5-triphosphate (PI(3,4,5)P_3_) (PIP3) lipids [45]. PIP3 is a key lipid second messenger that recruits and activates AKT, an essential component of the PI3K-AKT signaling pathway [23]. Our experiments show that higher levels of APOH enhance ferroptosis inhibition. A possible explanation is that sufficient APOH may cause the aggregation of PIP3 on the cell membrane, which in turn promotes the activation of the AKT signaling pathway. Notably, our findings reveal that deletion of domain V in APOH abrogates its ability to activate the PI3K-AKT signaling pathway and its ability to inhibit ferroptosis. More importantly, ApoHinfer, the integration of the two active regions within domain V responsible for phospholipid binding (AA 281–288) and insertion into the lipid membrane (AA 311–317), directly and effectively inhibits ferroptosis (Fig. 5F). These results emphasize the essential and sufficient role of domain V in APOH-mediated ferroptosis inhibition.

APOH plasma concentrations show a wide inter-individual variation, ranging from immunologically undetectable levels to concentrations of 350 μg/mL, with a mean of 150–200 μg/mL [46, 47]. The expression of APOH may be influenced by distinct physiological and pathological factors [32, 46, 48]. For example, alcohol downregulates *ApoH* expression, exacerbates fatty liver, and induces dysbiosis of lipid metabolism in a mouse model of alcoholic fatty liver disease (AFLD) [48]. Recent studies indicate that AFLD and non-alcoholic fatty liver disease (NAFLD) are caused by ferroptosis [4, 49]. These findings suggest that APOH levels are tightly regulated by physiological or pathological conditions and play an essential role in the regulation of ferroptosis. Increasing APOH levels or supplementing with ApoHinfer to alter cellular lipid metabolism could be a novel approach to preventing diseases associated with ferroptosis. Since ApoHinfer is a peptide derivative of APOH and APOH levels vary greatly between individuals, it is a potential candidate with minimal side effects that offers promising prospects for clinical application.

The level of APOH significantly influence the state of cells sensitive to ferroptosis, and a dose-dependent effect of APOH on activation of the AKT pathway was observed (Figs. 2F and 5A). Consistent with findings in this study, cancer cells harboring activating mutations in the AKT pathway and its downstream mTOR signaling pathway exhibit resistance to ferroptosis [50]. Patients with cancer bearing these mutations may be effectively treated with therapies that combine ferroptosis induction with mTOR inhibition [50]. Furthermore, it was discovered that patients with HCC with high APOH expression were less susceptible to sorafenib-induced ferroptosis. Our study implies that high serum APOH levels might confer resistance to sorafenib-induced ferroptosis, even in patients lacking activating mutations in the AKT pathway. Hence, considering serum APOH levels could be crucial in treating ferroptosis-related diseases.

SLC7A11 inhibits ferroptosis by facilitating cystine uptake for GSH synthesis. However, in HCC patients resistant to sorafenib, *SLC7A11* expression is lower. This discrepancy may be explained by the adaptation of cancer cells with low *SLC7A11* expression to reduced GSH levels in vivo. These cells likely develop compensatory mechanisms to maintain their antioxidant capacity, including the upregulation of *GPX4*, *APOH*, and *SCD* (Fig. 6B–D), with *GPX4* and *SCD* being well-known ferroptosis inhibitors. Consequently, HCC patients with low *SLC7A11* expression are resistant to inhibitors of this transporter by upregulating other antioxidant mechanisms.

We found that R-FBS also influenced the cholesterol biosynthesis pathway. Intermediate metabolites of the cholesterol biosynthesis pathway, such as CoQ [51, 52], squalene [53], and IPP [21], are involved in the regulation of ferroptosis, we also found that lovastatin, an inhibitor of cholesterol biosynthesis can partially abolished the effect of R-FBS (Fig. S5C, D). On the other hand, despite that IPP is essential for the efficient translation of GPX4 [20] and R-FBS affected the protein content of GPX4, the ability of R-FBS to inhibit ferroptosis was independent of the activity of GPX4 (Fig. S5F, G). Whether R-FBS influences ferroptosis through cholesterol biosynthesis pathway by CoQ or other metabolites requires further investigation.

In summary, this study highlights APOH as a novel extracellular regulator of the PI3K/AKT-SREBP1-SCD1 pathway, crucial for cellular resistance to ferroptosis by altering lipid composition. As serum plays a vital role in both in vivo and in vitro settings, this research underscores the tight regulation of ferroptosis within physiological or pathological conditions provided by serum. Moreover, it offers insights into establishing standard controls for serum usage in ferroptosis researches. Further investigations into additional serum proteins or small molecules modulating ferroptosis are warranted.

## Materials and methods

### Chemicals

RSL3 (B6095), Liproxstatin-1 (B4987), Fatostatin A (B5599), LY294002 (A8250), MK-2206 dihydrochloride (A3010), MK-8245 (A4345), Cell Counting Kit-8 (CCK-8, K1018) were purchased from APExBIO (TX, USA). Erastin2 (GC43623) was purchased from GlpBio (CA, USA). BODIPY 581/591 C11 (D3861) was purchased from Thermo Fisher (MA, USA).

### Cell culture

HEK 293T, MEF cell lines were acquired from the American Type Culture Collection. The Hepa 1–6, H1299, BT549, C2C12, CT26, 4T1, and SK-OV-3 cell lines were purchased from the Cell Resource Center, Shanghai Institutes for Biological Sciences, Chinese Academy of Sciences. All cell lines tested negative for Mycoplasma. Cell culture and all biological experiments were performed at 37 °C in 5% CO_2_ conditions in a cell culture incubator. HEK 293T, MEFs, and C2C12 cells were cultured in high-glucose Dulbecco’s modified Eagle’s medium (L110KJ, BasalMedia, Shanghai, China) supplemented with 10% indicated FBS, and 1% penicillin-streptomycin (S110JV, BasalMedia, Shanghai, China). H1299, BT549, and CT26 cell lines were cultured in RPMI 1640 (L210KJ, Basal Media, Shanghai, China) with the indicated FBS. SK-OV-3 cells were cultured in McCoy’s 5A medium (L630 KJ, Basal Media) supplemented with 10% FBS. Fetal bovine serum (FBS) used in this study was obtained from ExCell (FSP500, Lot No. 12A278; 12A327; 12B164; 12B172; 12J098; Taicang, China), Yeasen (40130ES76, Lot No. 504051820; 504062922, Shanghai, China), BioAgrio (S1370 and S1370L, USA), Sigma (F7524, USA), Thermo Fisher (30067334, USA), and Biological Industries (04-005-1A, Israel).

### Cell viability assay

MEFs, Hepa 1-6, H1299, BT549, C2C12, CT26, 4T1, and SK-OV-3 were seeded in 96-well plates at a density of 5 to 8 × 10^3^ cells/well for 24 h. Cells were treated with the compounds for 24 h, as indicated. The cells were exposed to 10 μL CCK-8 with 100 μL culture medium per well for 1–4 h in an incubator. Absorbance at 450 nm was measured using a microplate reader.

### Lipid peroxidation measurement

MEFs were seeded in 6-well plates at a density of 2 × 10^5^ cells/well and cultured in the indicated medium (Rcm or Scm). The next day, MEFs were treated with compounds for the indicated times before incubation with 2.5 μM of BODIPY 581/591 C11 for 30 min at 37 °C in a CO_2_ incubator. The cells were trypsinized, resuspended in the culture medium, and washed twice with phosphate-buffered saline (PBS). Lipid peroxidation was assessed using a flow cytometer with a 488 nm laser on an FL1 detector (Beckman CytoFlex S, Beckman, CA, USA). A minimum of 10,000 single cells per sample were analyzed.

### Ammonium sulfate precipitate and identification from FBS

All purification steps were performed at 4 °C. For purification, 110 mL R-FBS was applied to 50–90% saturation ammonium sulfate and incubated for 4 h. Each fraction was centrifuged at 12,000 × *g* for 20 min. The precipitate was resuspended and dialyzed overnight with Buffer A (20 mM HEPES [pH 7.5] and 50 mM NaCl). The mixture of 60–80% ASP fraction was loaded onto Q and SP Sepharose columns. The binding proteins were eluted with a gradient of 100–1000 mM NaCl in 20 mM HEPES (pH 7.5) and changed to Buffer A while concentrating with an Amicon Ultra-15 Centrifugal Filter (Millipore, 10 kDa cutoff). The fractions were separated by SDS-PAGE and examined using Coomassie Brilliant Blue staining. Protein concentrations were quantified using the Quick Start Bradford assay (Bio-Rad, CA, USA). And, the fractions were filtered with 0.22 μm filters and assayed for ferroptosis-inhibiting activity.

### Mass spectrometry

Equal amounts of protein from fractions of SP100–500 and Q300–500 were precipitated by adding methanol and chloroform (4:1, v/v) and then resuspended with 100 μL 100 mM TEAB (pH 7.5). Trypsin/Lys-C Mix (Promega, V5072) was added to protein at an enzyme-to-substrate ratio of 1:50 (w/w), and the digestion was conducted at 37 °C overnight. After digestion, the peptides were treated with 1% trifluoroacetic acid (TFA), purified using C18 Zip tips, and eluted with 0.1% TFA in 50–70% acetonitrile. The peptides were pre-processed and injected into an Orbitrap Fusion Lumos mass spectrometer (Thermo Scientific, Waltham, MA, USA) coupled with an EASY-nLC 1000 liquid chromatograph (Thermo Scientific, Waltham, MA, USA). Bioinformatics and statistical analyses of the original mass spectrometric (MS) data were performed using PEAKS Studio (version 8.5) with the Swiss-Prot database (updated November 2020). The *Bos taurus* database was used for the following result analysis. Only the hits with FDR ≤ 0.01 and the *P* ≤ 0.05 were accepted for discussion. The proteins of SP100-500 and Q300-500 identified by MS are listed in Table S1.

### APOH protein purification

The mouse Apoh cDNA was cloned from Core Facility of Basic Medical Sciences (Shanghai Jiao Tong University School of Medicine, SHSMU). PCR amplified using primers WT-APOH-F: 5′-TTGACACGAAGCTTGCCACCATGGTTTCCCCGGTGCTCGCCTTG-3′ and WT-APOH-R: 5′-TGAGATCCTCCAGGATCCATGCACGGTGTCAGTTCTGATG-3′; DDV-APOH-F: 5′-GATGGATCCTGGAGGATCTCATCATCACCACCACCATCAT-3′ and DDV-APOH-R: 5′-GAGATCCTCCAGGATCCATCTCTCTACAGGTCGGCAAGAA-3′. cDNAs encoding mouse WT-APOH and DDV-APOH tagged with 8×His were subcloned into the PKN plasmid obtained from Aiwu Zhou lab. HEK 293 T cells were transiently transfected using polyethylenimine (PEI; Polyscience, USA), and cells were cultured in 2% Scm for 48 h. The culture medium was harvested, and the insoluble substances were removed by 12,000 × *g* centrifugation for 15 min before filtration through a 0.22 μm filter. The medium with targeted protein was dialyzed overnight with Buffer B (20 mM HEPES [pH 7.5], 250 mM NaCl, and 20 mM imidazole) and then loaded onto HisTrap HP columns (Cytiva, 5 mL). After equilibrating the column with Buffer B, the targeted proteins were eluted with Buffer C (20 mM HEPES [pH 7.5], 250 mM NaCl, and 500 mM imidazole). Finally, the proteins were changed to Buffer A (20 mM HEPES [pH 7.5], 50 mM NaCl) while concentrating with an Amicon Ultra-15 Centrifugal Filter (Millipore, 10 kDa cutoff).

### ApoHinfer synthesis

ApoHinfer peptide (CKNKEKKCGGSSLAFWK) was purchased from ChinaPeptides Co, Ltd (QYAOBIO) and synthesized by 9-fluorenylmethyloxycarbonyl (Fmoc) solid phase peptide synthesis (SPPS) method. ApoHinfer was dissolved in PBS and added to the Scm at a suitable concentration for cell culture.

### Western blot

MEFs were seeded in 6-well plates (2.5 × 10^5^ cells/well) cultured in Scm for 24 h. For SCD1 detection, cultured media were replaced with the indicated media and for indicated times. To detect p-AKT, MEFs were seeded on 6-well plates at a density of 2.5 × 10^5^ cells/well and cultured in Scm for 24 h. The cells were then starved in serum-free DMEM for 12 h and treated with 10 µg/mL purified mouse WT-APOH or DDV-APOH protein diluted in serum-free DMEM for the indicated times. Cells were rinsed with ice-cold DPBS three times and lysed for 30 min at 4 °C using Mammalian Cell Lysis Buffer (MCLB): 50 mM Tris (pH 7.5), 150 mM NaCl, and 0.5% NP40, supplemented with 1 mM PMSF (Amresco, WA, USA), ethylenediaminetetraacetic acid-free protease inhibitor cocktail, and phosphatase inhibitor cocktail (Roche, Basel, Switzerland). Whole-cell proteins were obtained by 20 min centrifugation (13,000 × *g*, 4 °C), and protein concentrations were quantified using the Quick Start Bradford assay (Bio-Rad, CA, USA). The proteins were electrophoresed by 4%–20% SDS-PAGE using vertical electrophoresis (Tanon, Shanghai, China) and transferred to a nitrocellulose filter membrane (Millipore, MA, USA). The membranes were blocked with 5% defatted milk for 1 h and incubated with the indicated primary antibodies at 4 °C. Following 24 h, the membranes were washed thrice with Tris-buffered saline with Tween (TBST) (20 mmol/L Tris, pH 7.4; 137 mmol/L NaCl; 0.05% Tween-20) and then incubated with secondary antibodies (1:10,000 diluted in TBST) for 1 h at room temperature. Immunoreactive protein bands were detected by enhanced chemiluminescence using the ChampChemi imaging system (Sage Creation Science, Beijing, China). Anti-APOH (66074-1-Ig), anti-GPX4 (67763-1-Ig), anti-Albumin (16475-1-AP), anti-GAPDH (60004-1-Ig), anti-β-actin (66009-1-Ig), anti-tubulin (66031-1-Ig) and anti-His-tag (66005-1-Ig) were purchased from Proteintech (IL, USA). Anti-SCD1 (2794), anti-phospho-AKT Ser473 (9271 T), and anti-AKT (4691T) antibodies were purchased from Cell Signaling Technology (MA, USA). GAPDH and actin were used as internal controls.

### RNA-seq analysis and transcription factor prediction analysis

Total RNA was extracted from MEFs cultured in Scm and Rcm medium and from MEFs subjected to medium exchange for 12 h (R-Scm 12 h, S-Rcm 12 h) or 24 h (R-Scm 24 h, S-Rcm 24 h) using the RNeasy mini kit (Qiagen, Germany). RNA-Seq was performed at Sinotech Genomics Co., Ltd. (Shanghai, China). Library construction and sequencing were performed by Sinotech Genomics Co. Ltd. (Shanghai, China). Purified libraries were quantified by Qubit^®^ 2.0 Fluorometer (Life Technologies, USA) and validated by Agilent 2100 bioanalyzer (Agilent Technologies, USA), and sequenced on the Illumina NovaSeq 6000 (Illumina, USA). Paired-end sequence files (FASTQ) were mapped to the reference genome GRCm38.102 using Hista2 (Hierarchical Indexing for Spliced Alignment of Transcripts, version 2.0.5).

The DEGs between Rcm and Scm, Rcm and R-Scm 12 h, Rcm and R-Scm 24 h, Scm and S-Rcm 12 h, Scm and S-Rcm 24 h were analyzed using the DESeq2 package in R. DEGs were identified based on the following criteria: | log2 (fold change) | > 0.58 and adjust *P* < 0.05 across all groups. Significantly modulated mRNAs were selected for further analysis. KEGG pathway analysis of DEGs with “Clusterprofile” R package was performed.

### Bioinformatic analysis

Chip-sequencing library data was downloaded on the “Enrichr” website (https://maayanlab.cloud/Enrichr/) for transcription factor prediction analysis, and the R package “clusterProfiler” was used to predict the transcription factors of DEGs. The GSE109211 dataset was downloaded from the GEO database. GSE109211 was annotated using GPL13938, which included 21 sorafenib-responders and 46 sorafenib-non-responder samples. The “limma” R package was used to analyze the GSE109211 dataset.

### Real-time quantitative polymerase chain reaction (RT-qPCR)

Total RNA was isolated from MEFs using the EZ-press RNA Purification kit (B0004D, CA, USA) and then reverse transcribed into cDNAs using the HiScript III RT SuperMix for qPCR (with gDNA wiper) kit (R323-01, Vazyme, Nanjing, China). The corresponding cDNAs were subjected to qPCR analysis using SYBR Green Master Mix (11201ES08, YESEN, Shanghai, China) with a 384-plate well in a LightCycler480 apparatus (Roche, Basel, Switzerland). GAPDH was used as the internal control. Relative gene expression data were analyzed using the 2^−ΔΔCT^ method. The primers used for RT-qPCR assays are listed in Table S2.

### Lipidomics analysis

All lipidomic analyses, identification, and quality control (QC) were performed according to standard procedures carried out by BioTree (Shanghai, China). Briefly, MEFs cultured in Scm were switched to other media, including Scm, Rcm, 5% Scm + 5% Rcm, or 5% Scm + APOH (120 μg/mL), and incubated for 24 h. Cells were seeded at a density of ~70%–80% confluence at the time of extraction. Cell pellets collected in PBS were washed and centrifuged before freezing on dry ice. For each sample, 0.2 mL water and 0.48 mL extract solution [methyl tert-butyl ether/methanol = 5:1, (v/v)] were added sequentially. Samples were centrifuged at 900 × *g* for 15 min at 4 °C. Before reconstitution, the supernatant was transferred to a fresh tube and dried in 80 μL 50% methanol in dichloromethane. The composition was then centrifuged at 16000 × *g* for 15 min at 4 °C, and 100 μL of supernatant was transferred to a fresh glass vial for LC/MS analysis. The QC sample was prepared by mixing an equal aliquot 15 μL of the supernatants of all samples. LC-MS/MS analyses were performed using a UHPLC system (1290, Agilent Technologies) equipped with a Kinetex C18 column (2.1 × 100 mm, 1.7 μm, Phenomen). Mobile phase A comprised 40% water, 60% acetonitrile, and 10 mM ammonium formate. Mobile phase B consisted of 10% acetonitrile and 90% isopropanol, and 50 mL of 10 mM ammonium formate was added to every 1000 mL mixed solvent. The analysis was conducted with elution gradient as follows: 0~1 min, 40% B; 1~12 min, 40~100% B; 12~13 min, 100% B; 13.5~13.7 min, 100~40% B; 13.7~18 min, 40% B. The column temperature was set to 55 °C. The auto-sampler temperature was 4 °C, and the injection volume was 2 μL (pos) or 2 μL (neg). A mass spectrometer was used because of its ability to acquire MS/MS spectra in data-dependent acquisition (DDA) mode under the control of the acquisition software (Xcalibur 4.0.27, Thermo). The raw data files were converted to files in mzXML format using the “msconvert” program from ProteoWizard. The CentWave algorithm in XCMS was used for peak detection, extraction, alignment, and integration; the minfrac for annotation was set at 0.5, and the cutoff for annotation was set at 0.3. Lipid identification was achieved by spectral matching using the LipidBlast library, developed using R and based on XCMS. The final dataset containing information on peak number, sample name, and normalized peak area was imported into the SIMCA16.0.2 software package (Sartorius Stedim Data Analytics AB, Umea, Sweden) for multivariate analysis. To visualize group separation and identify significantly altered metabolites, supervised orthogonal projections to latent structures-discriminate analysis (OPLS-DA) were applied. The value of variable importance in the projection (VIP) of the first principal component of the OPLS-DA was obtained. This summarizes the contribution of each variable to the model. Lipids with VIP > 1 and *P* < 0.05 (Student’s t-test) were considered significantly changed.

### CRISPR knockout Gpx4

Single guide RNAs (sgRNA) were designed to target *Gpx4* in MEFs. The sgRNA targeting *Gpx4* 5′-caccgCATGCCCGATATGCTGAGTG-3′ was cloned into the lentiviral vector lenti-CRISPR-v2-Puro. The clone was transfected into HEK 293 T cells using a pMD2.G envelope plasmid, a psPAX2 packaging plasmid, and the transfection reagent PEI. After 48 h, the supernatant containing the lentivirus particles was collected for infection of MEFs. To enhance efficiency, 1 μg/ml polybrene was added simultaneously. Following 48 h infection period, MEFs were treated with 1 μg/ml puromycin (APExBIO, USA) for 3 days to select positive cells before single cells were sorted into 96-well plates. Single cells were maintained in complete medium with 5 μM Lip-1 for 4 weeks, and each colony was analyzed by western blotting to confirm *Gpx4* deletion.

### Statistics analysis and reproducibility

Statistical significance was assessed using two-tailed unpaired Student’s t-tests, with *P* values calculated using GraphPad Prism software. *P < 0.05, **P < 0.01, ***P < 0.001, ****P < 0.0001. NS, not significant. Data are presented as means ± standard deviation. The sample size was chosen based on the need for statistical power and was at least equal to the general sample size recommended in previous reports. The results of cell culture experiments were obtained from a minimum of three independent replicates. For immunoblots, the experiments were repeated at least three times with similar results, and representative data is shown, with the full and uncropped western blots provided in the Supplemental Material.

## Supplementary information

Supplement figures legends and tables

Original Western Blot

## Data Availability

The RNA-seq data have been deposited in GEO under the accession number GSE237178.

## References

[1] Conrad M, Pratt DA. The chemical basis of ferroptosis. Nat Chem Biol. 2019;15:1137–47. 10.1038/s41589-019-0408-1. 31740834 10.1038/s41589-019-0408-1

[2] Stockwell BR. Ferroptosis turns 10: emerging mechanisms, physiological functions, and therapeutic applications. Cell. 2022;185:2401–21. 10.1016/j.cell.2022.06.003. 35803244 10.1016/j.cell.2022.06.003 PMC9273022

[3] Ide S, Ide K, Abe K, Kobayashi Y, Kitai H, McKey J, et al. Sex differences in resilience to ferroptosis underlie sexual dimorphism in kidney injury and repair. Cell Rep. 2022;41:111610. 10.1016/j.celrep.2022.111610. 10.1016/j.celrep.2022.111610 PMC9795409 36351395

[4] Cui S, Ghai A, Deng Y, Li S, Zhang R, Egbulefu C, et al. Identification of hyperoxidized PRDX3 as a ferroptosis marker reveals ferroptotic damage in chronic liver diseases. Mol Cell. 2023;83:3931–9.e5. https://www.sciencedirect.com/science/article/pii/S1097276523007529. 37863053 10.1016/j.molcel.2023.09.025 PMC10841858

[5] Wu J, Minikes AM, Gao M, Bian H, Li Y, Stockwell BR, et al. Intercellular interaction dictates cancer cell ferroptosis via NF2–YAP signalling. Nature. 2019;572:402–6. 10.1038/s41586-019-1426-6. 31341276 10.1038/s41586-019-1426-6 PMC6697195

[6] Yang WS, Kim KJ, Gaschler MM, Patel M, Shchepinov MS, Stockwell BR. Peroxidation of polyunsaturated fatty acids by lipoxygenases drives ferroptosis. Proc Natl Acad Sci USA. 2016;113:E4966–75. 10.1073/pnas.1603244113. 27506793 10.1073/pnas.1603244113 PMC5003261

[7] Doll S, Proneth B, Tyurina YY, Panzilius E, Kobayashi S, Ingold I, et al. ACSL4 dictates ferroptosis sensitivity by shaping cellular lipid composition. Nat Chem Biol. 2017;13:91–8. 10.1038/nchembio.2239. 27842070 10.1038/nchembio.2239 PMC5610546

[8] Magtanong L, Ko P-J, To M, Cao JY, Forcina GC, Tarangelo A, et al. Exogenous monounsaturated fatty acids promote a ferroptosis-resistant cell state. Cell. Chem Biol. 2019;26:420–32.e9. https://www.sciencedirect.com/science/article/pii/S2451945618304380. 10.1016/j.chembiol.2018.11.016 PMC6430697 30686757

[9] Liu X, Olszewski K, Zhang Y, Lim EW, Shi J, Zhang X, et al. Cystine transporter regulation of pentose phosphate pathway dependency and disulfide stress exposes a targetable metabolic vulnerability in cancer. Nat Cell Biol. 2020;22:476–86. 10.1038/s41556-020-0496-x. 32231310 10.1038/s41556-020-0496-x PMC7194135

[10] Zhang W, Trachootham D, Liu J, Chen G, Pelicano H, Garcia-Prieto C, et al. Stromal control of cystine metabolism promotes cancer cell survival in chronic lymphocytic leukaemia. Nat Cell Biol. 2012;14:276–86. 10.1038/ncb2432. 22344033 10.1038/ncb2432 PMC3290742

[11] Yang WS, SriRamaratnam R, Welsch ME, Shimada K, Skouta R, Viswanathan VS, et al. Regulation of ferroptotic cancer cell death by GPX4. Cell. 2014;156:317–31. https://www.sciencedirect.com/science/article/pii/S0092867413015444. 24439385 10.1016/j.cell.2013.12.010 PMC4076414

[12] Armenta DA, Laqtom NN, Alchemy G, Dong W, Morrow D, Poltorack CD. et al.Ferroptosis inhibition by lysosome-dependent catabolism of extracellular protein.Cell Chem Biol. 2022;29:1588–1600.e7. https://www.sciencedirect.com/science/article/pii/S2451945622003609. 36306785 10.1016/j.chembiol.2022.10.006 PMC9762237

[13] Jiang X, Stockwell BR, Conrad M. Ferroptosis: mechanisms, biology, and role in disease. Nat Rev Mol Cell Biol. 2021;22:266–82. 10.1038/s41580-020-00324-8. 33495651 10.1038/s41580-020-00324-8 PMC8142022

[14] Duong-Ly KC, Gabelli SB. Chapter Seven - Salting out of proteins using ammonium sulfate precipitation. In: Lorsch JBT-M in E, editor. Laboratory methods in enzymology: protein part C. Academic Press; 2014. p. 85–94. https://www.sciencedirect.com/science/article/pii/B9780124201194000070. 10.1016/B978-0-12-420119-4.00007-0 24674064

[15] Leduc MS, Shimmin LC, Klos KLE, Hanis C, Boerwinkle E, Hixson JE. Comprehensive evaluation of apolipoprotein H gene (APOH) variation identifies novel associations with measures of lipid metabolism in GENOA. J Lipid Res. 2008;49:2648–56. 10.1194/jlr.M800155-JLR200. 18676959 10.1194/jlr.M800155-JLR200 PMC2582370

[16] Wang X, Sato R, Brown MS, Hua X, Goldstein JL. SREBP-1, a membrane-bound transcription factor released by sterol-regulated proteolysis. Cell. 1994;77:53–62. https://www.sciencedirect.com/science/article/pii/0092867494902348. 8156598 10.1016/0092-8674(94)90234-8

[17] Kamisuki S, Mao Q, Abu-Elheiga L, Gu Z, Kugimiya A, Kwon Y, et al. A small molecule that blocks fat synthesis by inhibiting the activation of SREBP. Chem Biol. 2009;16:882–92. https://www.sciencedirect.com/science/article/pii/S1074552109002385. 19716478 10.1016/j.chembiol.2009.07.007

[18] Wang C, Shi M, Ji J, Cai Q, Zhao Q, Jiang J, et al. Stearoyl-CoA desaturase 1 (SCD1) facilitates the growth and anti-ferroptosis of gastric cancer cells and predicts poor prognosis of gastric cancer. Aging. 2020;12:15374–91. http://europepmc.org/abstract/MED/32726752. 32726752 10.18632/aging.103598 PMC7467382

[19] Friedmann Angeli JP, Schneider M, Proneth B, Tyurina YY, Tyurin VA, Hammond VJ, et al. Inactivation of the ferroptosis regulator Gpx4 triggers acute renal failure in mice. Nat Cell Biol. 2014;16:1180–91. 10.1038/ncb3064. 25402683 10.1038/ncb3064 PMC4894846

[20] Warner GJ, Berry MJ, Moustafa ME, Carlson BA, Hatfield DL, Faust JR. Inhibition of selenoprotein synthesis by selenocysteine tRNA[Ser]Sec lacking isopentenyladenosine. J Biol Chem. 2000;275:28110–9. https://www.sciencedirect.com/science/article/pii/S0021925819650921. 10821829 10.1074/jbc.M001280200

[21] Viswanathan VS, Ryan MJ, Dhruv HD, Gill S, Eichhoff OM, Seashore-Ludlow B, et al. Dependency of a therapy-resistant state of cancer cells on a lipid peroxidase pathway. Nature. 2017;547:453–7. 10.1038/nature23007. 28678785 10.1038/nature23007 PMC5667900

[22] Jiang S-Y, Li H, Tang J-J, Wang J, Luo J, Liu B, et al. Discovery of a potent HMG-CoA reductase degrader that eliminates statin-induced reductase accumulation and lowers cholesterol. Nat Commun. 2018;9:5138. 10.1038/s41467-018-07590-3. 30510211 10.1038/s41467-018-07590-3 PMC6277434

[23] Di-Luoffo M, Ben-Meriem Z, Lefebvre P, Delarue M, Guillermet-Guibert J. PI3K functions as a hub in mechanotransduction. Trends Biochem Sci. 2021;46:878–88. https://www.sciencedirect.com/science/article/pii/S0968000421001080. 34112586 10.1016/j.tibs.2021.05.005

[24] Krycer JR, Sharpe LJ, Luu W, Brown AJ. The Akt–SREBP nexus: cell signaling meets lipid metabolism. Trends Endocrinol Metab. 2010;21:268–76. https://www.sciencedirect.com/science/article/pii/S1043276010000032. 20117946 10.1016/j.tem.2010.01.001

[25] Romani P, Brian I, Santinon G, Pocaterra A, Audano M, Pedretti S, et al. Extracellular matrix mechanical cues regulate lipid metabolism through Lipin-1 and SREBP. Nat Cell Biol. 2019;21:338–47. 10.1038/s41556-018-0270-5. 30718857 10.1038/s41556-018-0270-5

[26] Wu K, Yan M, Liu T, Wang Z, Duan Y, Xia Y, et al. Creatine kinase B suppresses ferroptosis by phosphorylating GPX4 through a moonlighting function. Nat Cell Biol. 2023;25:714–25. 10.1038/s41556-023-01133-9. 37156912 10.1038/s41556-023-01133-9

[27] Hao J, Chen Q, Feng Y, Jiang Q, Sun H, Deng B, et al. Combination treatment with FAAH inhibitors/URB597 and ferroptosis inducers significantly decreases the growth and metastasis of renal cell carcinoma cells via the PI3K-AKT signaling pathway. Cell Death Dis. 2023;14:247. 10.1038/s41419-023-05779-z. 37024452 10.1038/s41419-023-05779-z PMC10079857

[28] Sanghera DK, Wagenknecht DR, McIntyre JA, Kamboh MI. Identification of structural mutations in the fifth domain of apolipoprotein H (β2-Glycoprotein I) which affect phospholipid binding. Hum Mol Genet. 1997;6:311–6. 10.1093/hmg/6.2.311. 9063752 10.1093/hmg/6.2.311

[29] Schwarzenbacher R, Zeth K, Diederichs K, Gries A, Kostner GM, Laggner P, et al. Crystal structure of human β2‐glycoprotein I: implications for phospholipid binding and the antiphospholipid syndrome. EMBO J. 1999;18:6228–39. 10.1093/emboj/18.22.6228. 10562535 10.1093/emboj/18.22.6228 PMC1171686

[30] Wang F, Xia X-F, Sui S. Human apolipoprotein H may have various orientations when attached to lipid layer. Biophys J. 2002;83:985–93. https://www.sciencedirect.com/science/article/pii/S0006349502752247. 12124280 10.1016/S0006-3495(02)75224-7 PMC1302202

[31] Castro, Lázaro A, Selva I, Céspedes DM, Girona E, NúriaPlana J, et al. APOH is increased in the plasma and liver of type 2 diabetic patients with metabolic syndrome. Atherosclerosis. 2010;209:201–5. 10.1016/j.atherosclerosis.2009.09.072. 19878946 10.1016/j.atherosclerosis.2009.09.072

[32] Okubo S, Miyamoto M, Ito D, Takami K, Ashida K. Albumin and apolipoprotein H mRNAs in human plasma as potential clinical biomarkers of liver injury: analyses of plasma liver-specific mRNAs in patients with liver injury. Biomarkers. 2016;21:353–62. 10.3109/1354750X.2016.1141987. 26901698 10.3109/1354750X.2016.1141987

[33] Di Maio M, Daniele B, Perrone F. Role of sorafenib in HCC patients with compromised liver function. Nat Rev Clin Oncol. 2009;6:505–6. 10.1038/nrclinonc.2009.114. 19707242 10.1038/nrclinonc.2009.114

[34] Dixon SJ, Patel DN, Welsch M, Skouta R, Lee ED, Hayano M, et al. Pharmacological inhibition of cystine-glutamate exchange induces endoplasmic reticulum stress and ferroptosis. eLife. 2014;3:e02523. http://europepmc.org/abstract/MED/24844246. 10.7554/eLife.02523 PMC4054777 24844246

[35] Yuan S, Wei C, Liu G, Zhang L, Li J, Li L, et al. Sorafenib attenuates liver fibrosis by triggering hepatic stellate cell ferroptosis via HIF-1α/SLC7A11 pathway. Cell Prolif. 2022;55:e13158. 10.1111/cpr.13158. 34811833 10.1111/cpr.13158 PMC8780895

[36] Zhang L, Li X, Shi X, Ye K, Fu X, Wang X, et al. Sorafenib triggers ferroptosis via inhibition of HBXIP/SCD axis in hepatocellular carcinoma. Acta Pharmacol Sin. 2023;44:622–34. 10.1038/s41401-022-00981-9. 36109580 10.1038/s41401-022-00981-9 PMC9958095

[37] Pinyol R, Montal R, Bassaganyas L, Sia D, Takayama T, Chau G-Y, et al. Molecular predictors of prevention of recurrence in HCC with sorafenib as adjuvant treatment and prognostic factors in the phase 3 STORM trial. Gut. 2019;68:1065–75. http://gut.bmj.com/content/68/6/1065.abstract. 30108162 10.1136/gutjnl-2018-316408 PMC6580745

[38] Gao M, Monian P, Quadri N, Ramasamy R, Jiang X. Glutaminolysis and transferrin regulate ferroptosis. Mol Cell. 2015;59:298–308. https://www.sciencedirect.com/science/article/pii/S1097276515004505. 26166707 10.1016/j.molcel.2015.06.011 PMC4506736

[39] Gao M, Yi J, Zhu J, Minikes AM, Monian P, Thompson CB, et al. Role of mitochondria in ferroptosis. Mol Cell. 2019;73:354–63.e3. https://www.sciencedirect.com/science/article/pii/S1097276518309365. 30581146 10.1016/j.molcel.2018.10.042 PMC6338496

[40] Zhang Y, Swanda RV, Nie L, Liu X, Wang C, Lee H. et al. mTORC1 couples cyst(e)ine availability with GPX4 protein synthesis and ferroptosis regulation. Nat Commun. 2021;12:1589.10.1038/s41467-021-21841-w. 33707434 10.1038/s41467-021-21841-w PMC7952727

[41] Chen Z, Ho I-L, Soeung M, Yen E-Y, Liu J, Yan L, et al. Ether phospholipids are required for mitochondrial reactive oxygen species homeostasis. Nat Commun. 2023;14:2194.10.1038/s41467-023-37924-9. 37069167 10.1038/s41467-023-37924-9 PMC10110566

[42] Mehta A, Shapiro MD. Apolipoproteins in vascular biology and atherosclerotic disease. Nat Rev Cardiol. 2022;19:168–79. 10.1038/s41569-021-00613-5. 34625741 10.1038/s41569-021-00613-5

[43] Niessen HWM, Lagrand WK, Rensink HJAM, Meijer CJLM, Aarden L, Hack CE. Apolipoprotein H, a new mediator in the inflammatory changes ensuing in jeopardised human myocardium. J Clin Pathol. 2000;53:863–7. http://jcp.bmj.com/content/53/11/863.abstract. 11127271 10.1136/jcp.53.11.863 PMC1731112

[44] Kamboh MI, Mehdi H. Genetics of apolipoprotein H (β2-glycoprotein I) and anionic phospholipid binding. Lupus. 1998;7:10–3. 10.1177/096120339800700203. 10.1177/096120339800700203 9814664

[45] Bidlingmaier S, Liu B. Interrogating yeast surface-displayed human proteome to identify small molecule-binding proteins. Mol Cell Proteomics. 2007;6:2012–20. 10.1074/mcp.M700223-MCP200. 17660511 10.1074/mcp.M700223-MCP200

[46] Mehdi H, Aston CE, Sanghera DK, Hamman RF, Kamboh MI. Genetic variation in the apolipoprotein H (β2-glycoprotein I) gene affects plasma apolipoprotein H concentrations. Hum Genet. 1999;105:63–71. 10.1007/s004399900089. 10480357 10.1007/s004399900089

[47] Willems GM, Janssen MP, Pelsers MMAL, Comfurius P, Galli M, Zwaal RFA, et al. Role of divalency in the high-affinity binding of anticardiolipin antibody−β2-glycoprotein I complexes to lipid membranes. Biochemistry. 1996;35:13833–42. 10.1021/bi960657q. 8901526 10.1021/bi960657q

[48] Liu Y, Wu Z, Zhang Y, Chen B, Yu S, Li W, et al. Alcohol-dependent downregulation of apolipoprotein H exacerbates fatty liver and gut microbiota dysbiosis in mice. Lipids Health Dis. 2022;21:89. 10.1186/s12944-022-01699-7. 36123743 10.1186/s12944-022-01699-7 PMC9487114

[49] You Y, Liu C, Liu T, Tian M, Wu N, Yu Z, et al. FNDC3B protects steatosis and ferroptosis via the AMPK pathway in alcoholic fatty liver disease. Free Radic Biol Med. 2022;193:808–19. https://www.sciencedirect.com/science/article/pii/S0891584922009534. 36336231 10.1016/j.freeradbiomed.2022.10.322

[50] Yi J, Zhu J, Wu J, Thompson CB, Jiang X. Oncogenic activation of PI3K-AKT-mTOR signaling suppresses ferroptosis via SREBP-mediated lipogenesis. Proc Natl Acad Sci USA. 2020;117:31189–97. 10.1073/pnas.2017152117. 33229547 10.1073/pnas.2017152117 PMC7733797

[51] Doll S, Freitas FP, Shah R, Aldrovandi M, da Silva MC, Ingold I, et al. FSP1 is a glutathione-independent ferroptosis suppressor. Nature. 2019;575:693–8. 10.1038/s41586-019-1707-0. 31634899 10.1038/s41586-019-1707-0

[52] Shimada K, Skouta R, Kaplan A, Yang WS, Hayano M, Dixon SJ, et al. Global survey of cell death mechanisms reveals metabolic regulation of ferroptosis. Nat Chem Biol. 2016;12:497–503. 27159577 10.1038/nchembio.2079 PMC4920070

[53] Garcia-Bermudez J, Baudrier L, Bayraktar EC, Shen Y, La K, Guarecuco R, et al. Squalene accumulation in cholesterol auxotrophic lymphomas prevents oxidative cell death. Nature. 2019;567:118–22. 10.1038/s41586-019-0945-5. 30760928 10.1038/s41586-019-0945-5 PMC6405297

