# Correction: Serum apolipoprotein H determines ferroptosis resistance by modulating cellular lipid composition

**DOI:** 10.1038/s41419-024-07181-9

**Published:** 2024-12-03

**Authors:** Xiang He, Jiahui Zhang, Masha Huang, Jie Wang, Simin Yang, Xiang Yu, Yingjie Xu, Wen Yang

**Affiliations:** 1 Department of Biochemistry and Molecular Cell Biology, Shanghai Key Laboratory for Tumor Microenvironment and Inflammation, Shanghai Jiao Tong University School of Medicine, Shanghai, China; 2 Core Facility of Basic Medical Sciences, Shanghai Jiao Tong University School of Medicine, Shanghai, China

**Keywords:** Necroptosis, Lipid peroxides

Correction to: *Cell Death & Disease* 10.1038/s41419-024-07099-2, published online 01 October 2024

In this article the Figs. 1H, I and 5A have been given erroneously due to a conversion mistake during the production process.

The correct figures are given below.
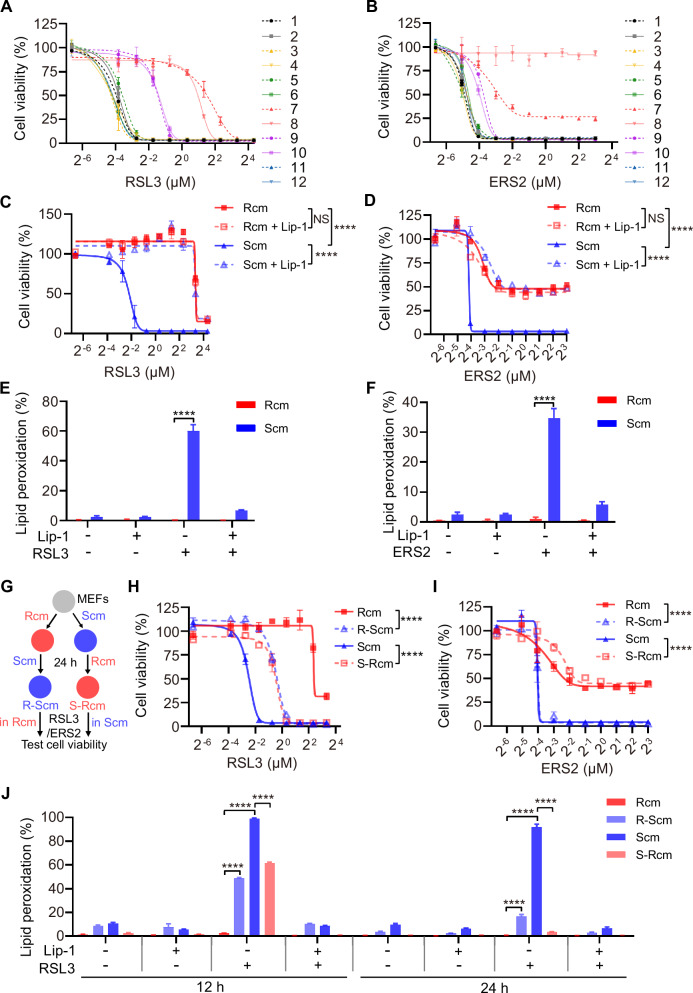

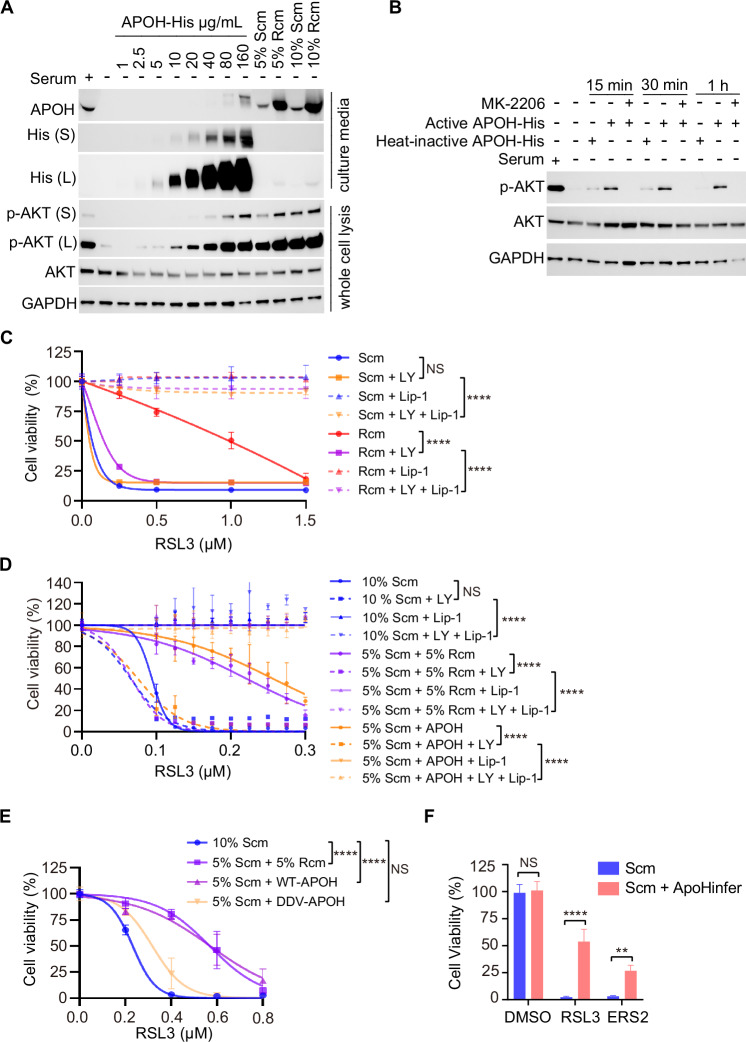


The original article has been corrected.

