# Supplementary material for: Serum apolipoprotein H determines ferroptosis resistance by modulating cellular lipid composition
Source: Cell Death Dis. 2024 Oct 1;15(10):718. doi: 10.1038/s41419-024-07099-2 (PMC11445452; doi:10.1038/s41419-024-07099-2)
Supplement: Supplementary file 1 — Supplement figures legends and tables [file 41419_2024_7099_MOESM1_ESM.docx]

**Supplementary Information for**

**Serum Apolipoprotein H Determines Ferroptosis Resistance by Modulating Cellular Lipid Composition**

Xiang He^1, #^, Jiahui Zhang^1, #^, Masha Huang^1, #^, Jie Wang^1^, Simin Yang^2^, Xiang Yu^1^, Yingjie Xu^1^, Wen Yang^1, *^

^1^Department of Biochemistry and Molecular Cell Biology, Shanghai Key Laboratory for Tumor Microenvironment and Inflammation, Shanghai Jiao Tong University School of Medicine, Shanghai 200025, China

^2^Core Facility of Basic Medical Sciences, Shanghai Jiao Tong University School of Medicine, Shanghai 200025, China

^#^These authors contributed equally: Xiang He, Jiahui Zhang, Masha Huang.

^*^Correspondence to Wen Yang.

Email: yangwen@shsmu.edu.cn

Supplementary Figure 1-7

Supplementary Table 1-2

**Figures**

**Fig. S1**

**
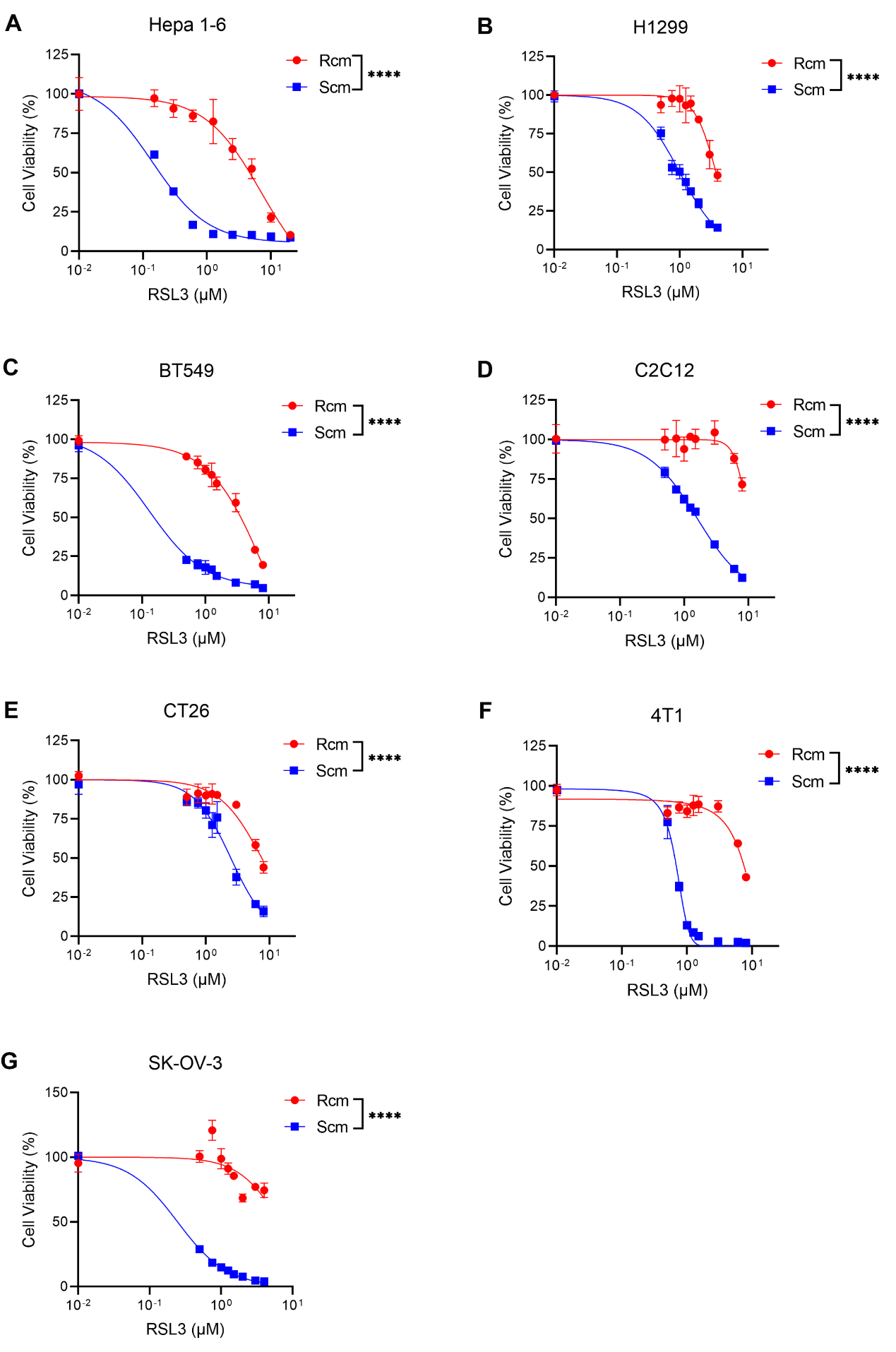
**

**Fig. S1. Serum changes the sensitivity of cells to ferroptosis.**

**(A-G)** Cell Viability of Hepa 1-6 **(A)**, H1299 **(B)**, BT549 **(C)**, C2C12 **(D)**, CT26 **(E)**, 4T1 **(F)**, and SK-OV-3 **(G)** cells cultured in Rcm or Scm and treated with different doses of RSL3. Data are presented as mean ± s.d., *n* = 3 independent repeats. Unpaired, two-tailed *t*-test; *****P* < 0.0001.

**Fig. S2**

**
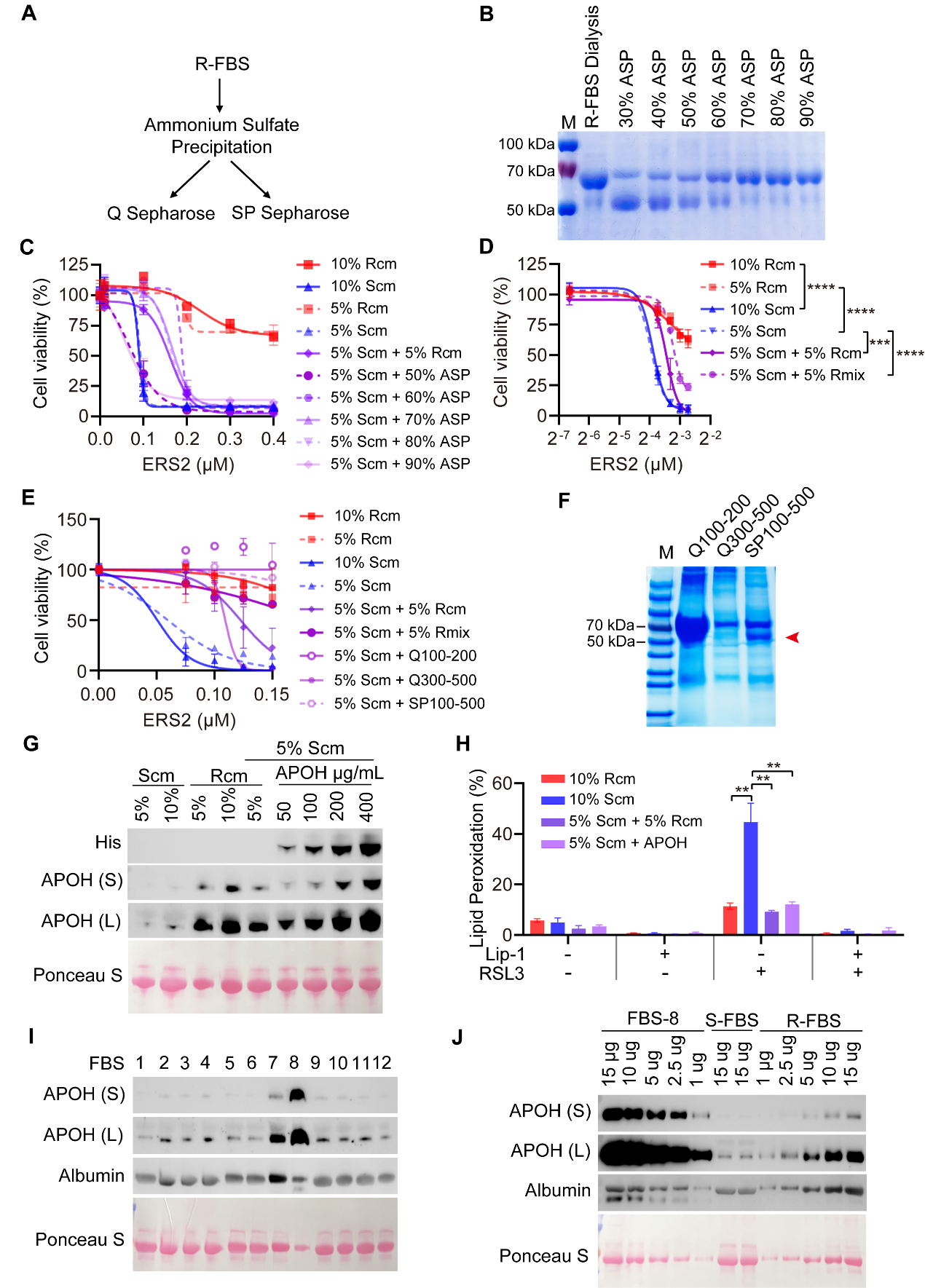
**

**Fig. S2. Serum protein APOH inhibits ferroptosis.**

**(A)** Procedure for the purification of the ferroptosis-inhibiting proteins of R-FBS. **(B)** Coomassie brilliant blue staining of the same protein amounts of fractions of 30–90% ammonium sulfate precipitated (ASP) of R-FBS. The amount of protein in dialyzed R-FBS and resuspended 30–90% ASP was quantified using the Bradford method. **(C)** Cell viability of MEFs cultured in Scm was assessed after switching to 5% or 10% Rcm or Scm, or complete medium containing 5% S-FBS with the addition of various components in equal protein amounts, including 5% R-FBS and fractions of 50% to 90% ASP of R-FBS, for 24 h, followed by treatment with the indicated concentration of ERS2 for 24 h in Scm. **(D)** Cell viability of MEFs cultured in Scm changed to 5% or 10% of Rcm or Scm, or complete medium containing 5% S-FBS with the addition of 5% R-FBS or 5% Rmix for 24 h and treated with the indicated concentration of ERS2 for 24 h in Scm. Rmix, the mixture of 60–80% ASP of R-FBS. **(E)** Cell viability of MEFs cultured in Scm changed to 5% or 10% of Rcm or Scm, or medium containing 5% S-FBS with the addition of various components, including with 5% R-FBS, 5% Rmix, Q100-200, Q300-500, or SP100-500 for 24 h and treated with the indicated concentration of ERS2 for 24 h in Scm. **(F)** Coomassie brilliant blue of Rmix purified by HiTrap-Q (Q) or HiTrap-SP (SP) column chromatography. Lane Q100-200, Q300-500 loaded with Rmix eluted with 100–200 mM or 300–500 mM NaCl in Q column chromatography; lane of SP100-500 loaded with Rmix eluted with 100–500 mM NaCl in SP column chromatography. The red arrow indicates the protein band located between 50 kDa and 70 kDa. **(G)** Western blot analysis of the protein levels of APOH tagged with His in the culture medium. Ponceau S staining was used as the loading control. **(H)** Lipid peroxidation in MEFs treated with RSL3. MEFs were cultured in Scm, Rcm, 5% Scm + 5% Rcm, or 5% Scm + APOH (120 μg/mL) for 24 h. Subsequently, MEFs treated with RSL3 (2 μM) with or without Lip-1(1 μM) for 2 h as indicated before staining with C11 (2.5 μM) for 30 min and tested by flow cytometry. **(I)** Western blot analysis of APOH protein levels in equal amounts of FBS-1 to FBS-12. Ponceau S staining was used as the loading control. **(J)** Western blotting analysis of APOH protein levels in FBS-8, S-FBS (FBS-1), and R-FBS (FBS-7). Ponceau S staining was used as the loading control. S, short exposure; L, long exposure. Western blotting and Ponceau S staining are representatives of three biological replicates (**B, F, G, I, J**). Data are presented as mean ± s.d., *n* = 3 independent repeats. Unpaired, two-tailed *t*-test; ***P* < 0.01, ****P* < 0.001, *****P* < 0.0001.

**Fig. S3**


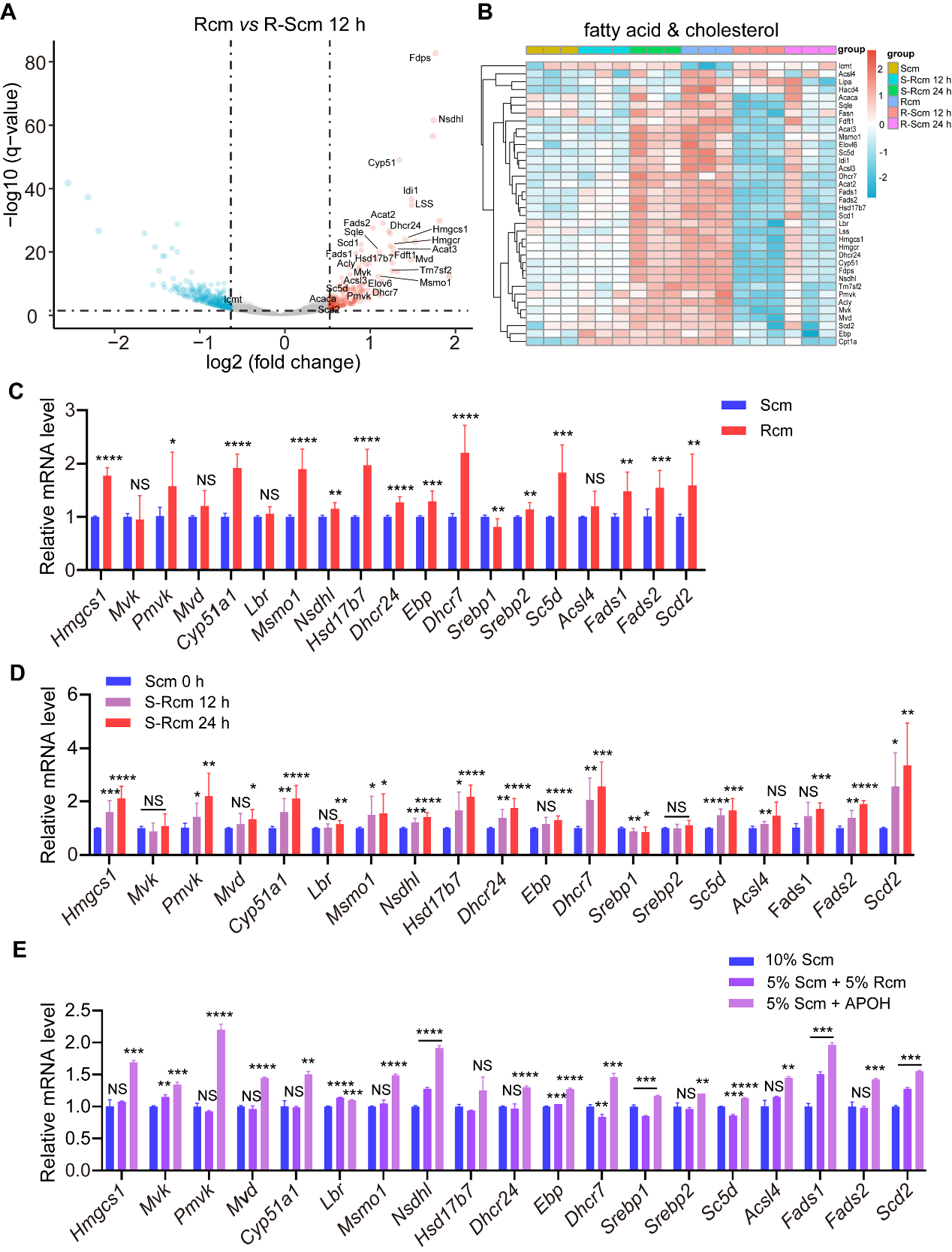


**Fig. S3. APOH increased the fatty acid biosynthesis pathway.**

**(A)** Volcano plot of DEGs related to the fatty acid and cholesterol biosynthesis pathways in MEFs cultured in Rcm and R-Scm 12 h medium. **(B)** Heat map showing DEGs of fatty acid and cholesterol biosynthesis pathways. MEFs were cultured in Rcm, Scm, and/or changed medium for 12 h and 24 h. **(C)** mRNA levels of the indicated SREBP1 target genes in MEFs cultured in Scm or Rcm were determined using RT-qPCR. **(D)** mRNA levels of the indicated SREBP1 target genes of MEFs cultured in Scm with or without medium change to Rcm for 12 h or 24 h were analyzed by RT-qPCR. **(E)** mRNA level of the indicated SREBP1 target genes of MEFs cultured in Scm changed medium to 5% Scm or 5% Scm + 5% Rcm, or 5% Scm + APOH (120 μg/mL) for 12 h were analyzed by RT-qPCR. Data are presented as mean ± s.d., *n* = 3 independent repeats. Unpaired, two-tailed *t*-test; **P* < 0.05, ***P* < 0.01, ****P* < 0.001, *****P* < 0.0001. NS, not significant.

**Fig. S4**


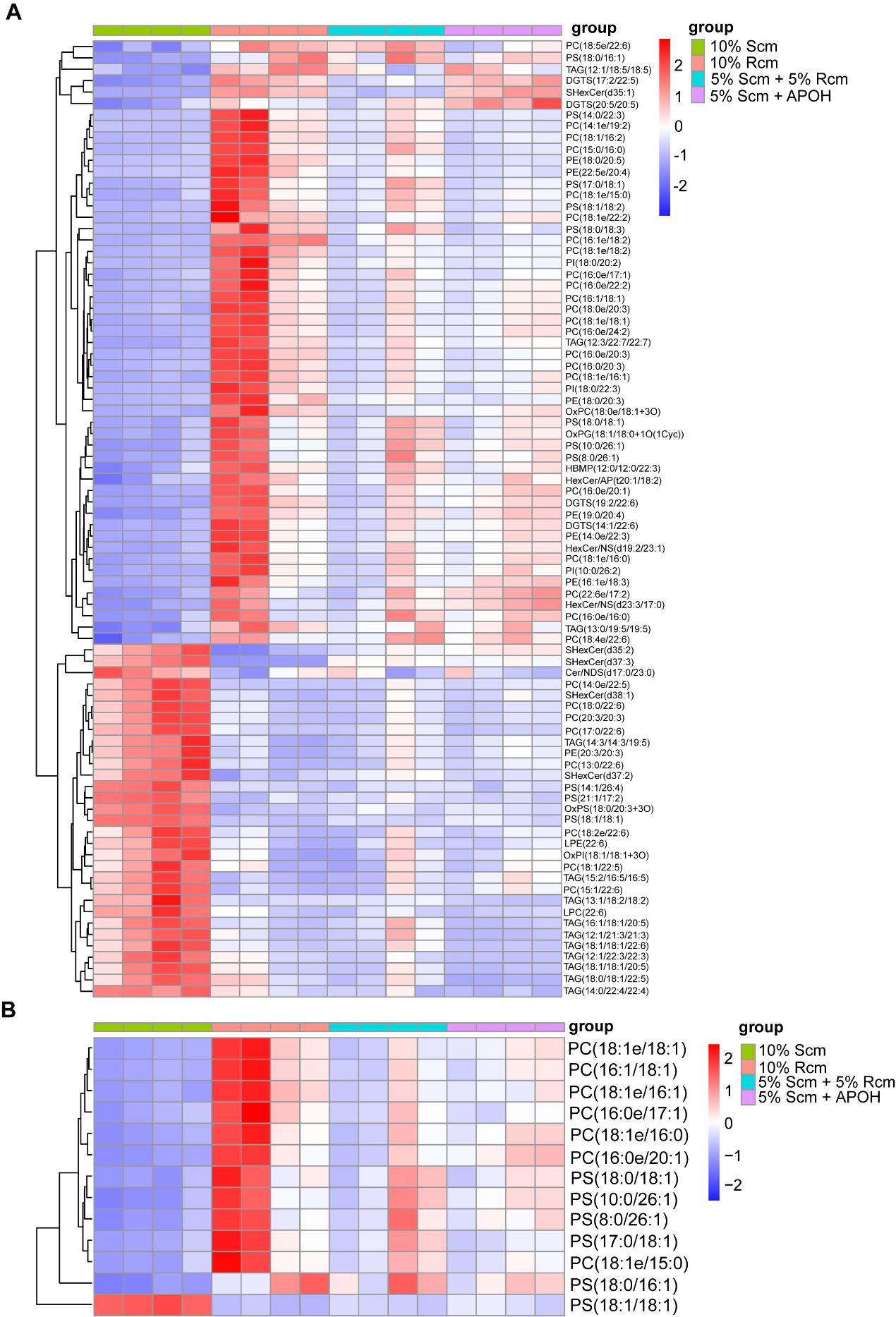


**Fig. S4. APOH alters cellular lipid composition.**

**(A, B)** Heatmap analysis to visualize the clustering of 84 differentially altered lipids **(A)** and to visualize the clustering of MUFA-PLs/MUFA-ePLs of MEFs **(B)** cultured in Scm, Rcm, 5% Scm + 5% Rcm, or 5% Scm + APOH (120 μg/mL). Four replicates were used for each condition. Each lipid species was normalized to its corresponding mean value.

**Fig. S5**


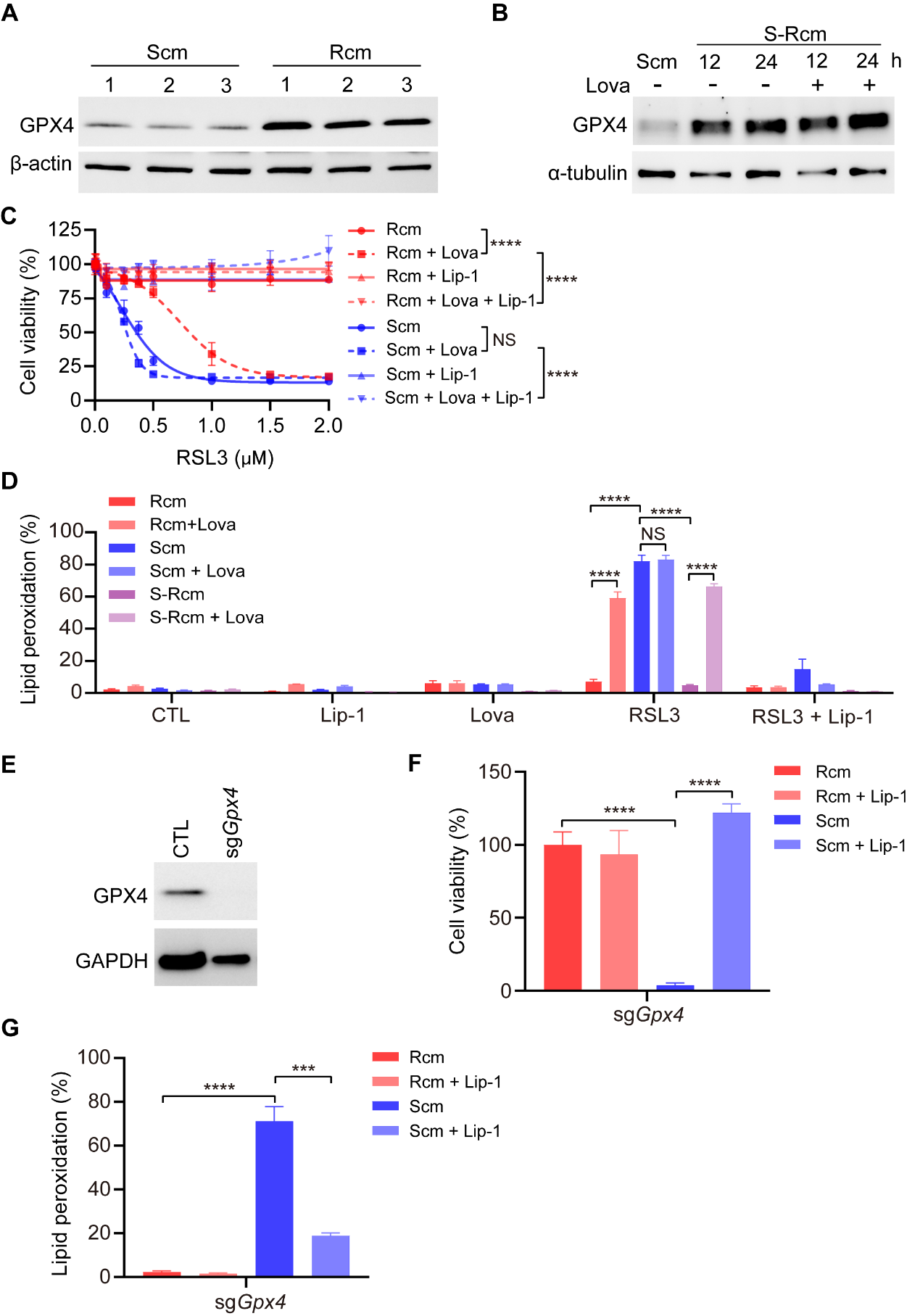


**Fig. S5. R-FBS resistance to ferroptosis is independent of the GPX4 activity.**

**(A)** Western blot analysis of GPX4 in MEFs cultured in Scm and Rcm media (*n* = 3 per group). **(B)** Western blot analysis of GPX4 of MEFs cultured in Scm or changed medium to Rcm with or without Lova (3 μM). S-Rcm, the culture medium of MEFs was changed from Scm to Rcm for 12 h or 24 h. **(C)** Cell viability of MEFs cultured in Scm changed medium to Rcm or not, treated with different doses of RSL3 and/or Lip-1 (1 μM) for 24 h following pretreatment with Lova (3 μM) for 24 h. **(D)** Lipid peroxidation of MEFs cultured in Scm changed medium to Rcm or not, treated with RSL3 (2 μM) and/or Lip-1 (1 μM) for 24 h following pretreatment with Lova (3 μM) for 24 h. **(E)** Western blot analysis of GPX4 protein levels in control and sg*Gpx4* knockout (KO) MEFs. **(F, G)** Cell viability **(F)** and lipid peroxidation **(G)** of sg*Gpx4* KO MEFs cultured in Rcm or Scm with or without Lip-1 (1 μM). Western blotting results are representative of three biological replicates. Data are presented as mean ± s.d., *n* = 3 independent repeats. Unpaired, two-tailed *t*-test; ****P* < 0.001, *****P* < 0.0001. NS, not significant.

**Fig. S6**


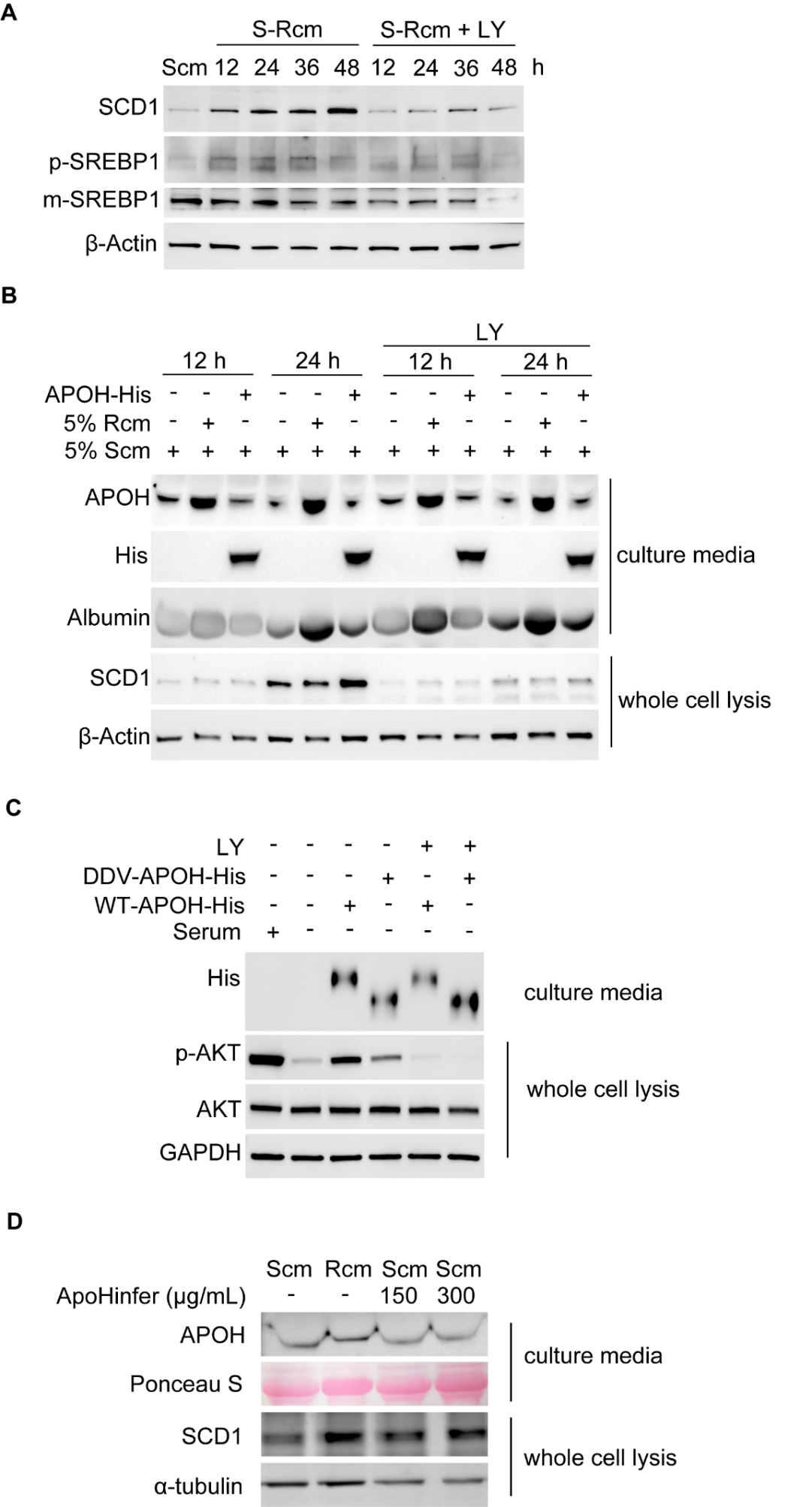


**Fig. S6. APOH inhibits ferroptosis by activating the AKT-SREBP1-SCD pathway.**

**(A)** Western blot analysis of SCD1 and SREBP1 protein levels in MEFs cultured in Scm and in cells in which the medium was changed from Scm to Rcm for the indicated times with or without LY (12 μM). **(B)** Western blot analysis protein levels of SCD1 in MEFs cultured in Scm and in cells in which the medium was changed from Scm to 5% Scm or 5% Scm + 5% Rcm, or 5% Scm + APOH (120 μg/mL) for the indicated time with or without LY (12 μM). **(C)** Western blot analysis was performed to detect p-AKT and AKT of MEFs and the His-tag of His in the culture media. The MEFs cultured in Scm were serum-starved for 12 h and then treated with the 10 μg/mL WT-APOH or the DDV-APOH with or without LY (12 μM) for 30 min. DDV-APOH-His, the deleted domain V mutant variant of APOH (DDV-APOH) tagged with His. WT-APOH-His, wild-type APOH tagged with His. **(D)** Western blot analysis protein levels of SCD1 in MEFs cultured in Scm and in cells in which the medium was changed from Scm to Rcm, or Scm adding ApoHinfer (150 μg/mL or 300 μg/mL) for 24 h. Western blotting is representative of three biological replicates **(A-D)**.

**Fig. S7**


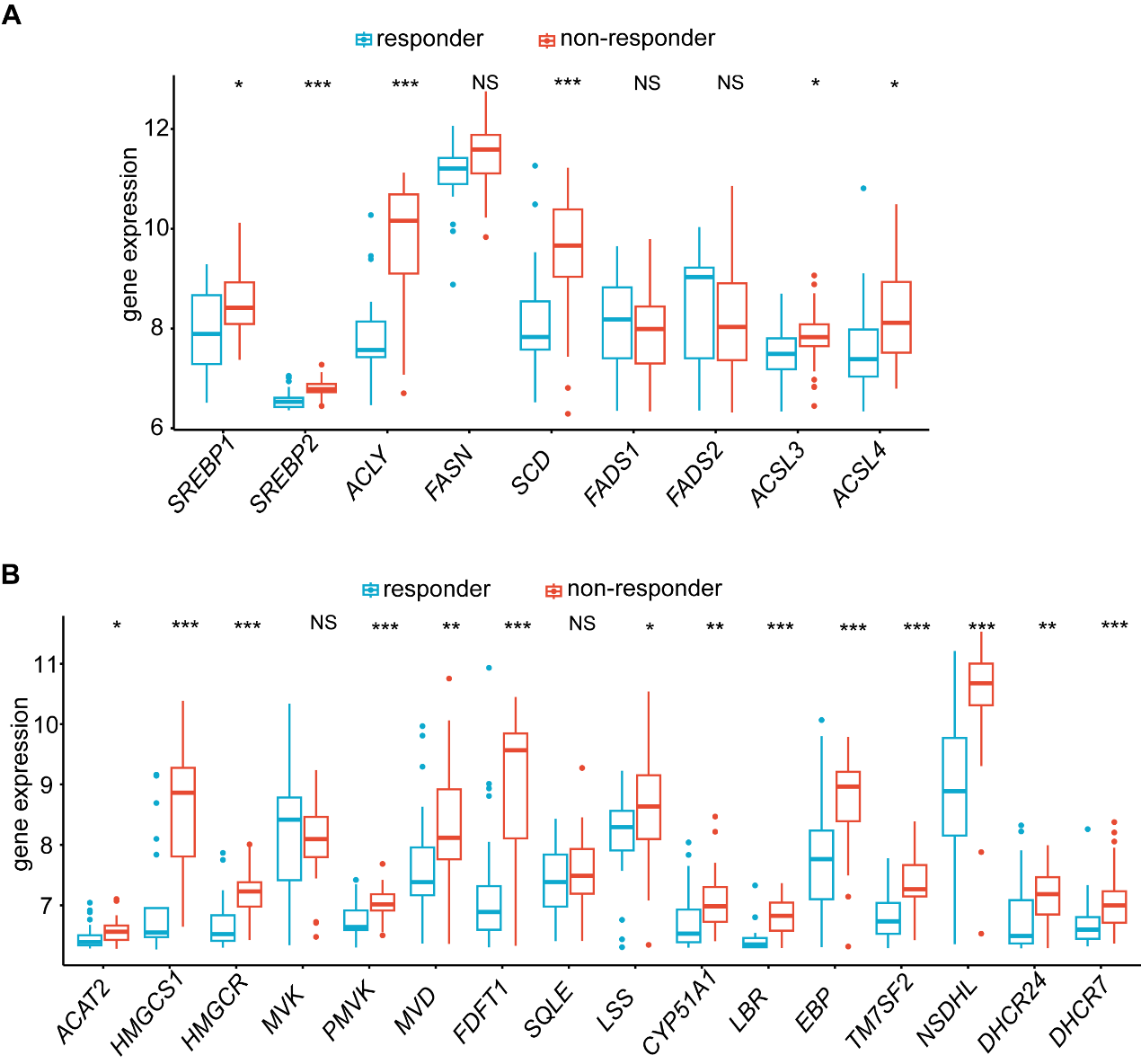


**Fig. S7.** **Genes expression of fatty acid biosynthesis and cholesterol biosynthesis pathway association with sorafenib response in patients with HCC.**

**(A, B)** Expression of fatty acid biosynthesis pathway **(A)** and cholesterol biosynthesis pathway **(B)** genes in HCC from 21 sorafenib responders and 46 non-responders in the GEO dataset (GSE109211). Statistical differences between the two groups were compared using a *t*-test. **P* < 0.05, ***P* < 0.01, ****P* < 0.001. NS, not significant.

| **Table S1. Proteins of SP100-500 and Q300-500 identified by MS, and the intensity ratio of SP100-500 to Q300-500.** | | | |
| --- | --- | --- | --- |
| **UniProt ID** | **Intensity of SP100-500** | **Intensity of Q300-500** | **Ratio of SP100-500 to Q300-500** |
| Q2KIG3 | 4.26E+09 | 8.64E+06 | 493.0555556 |
| Q95121 | 2.32E+10 | 1.18E+08 | 196.6101695 |
| Q2KJ63 | 2.05E+09 | 1.39E+07 | 147.4820144 |
| P17690 | 9.59E+10 | 9.05E+08 | 105.9668508 |
| P98140 | 5.36E+09 | 5.57E+07 | 96.22980251 |
| Q28085 | 1.66E+11 | 1.92E+09 | 86.45833333 |
| Q2KIS7 | 1.22E+10 | 1.85E+08 | 65.94594595 |
| Q9N2I2 | 1.99E+09 | 3.51E+07 | 56.6951567 |
| P41361 | 1.97E+09 | 5.50E+07 | 35.81818182 |
| P63258 | 1.50E+09 | 5.04E+07 | 29.76190476 |
| P68103 | 6.24E+07 | 2.81E+06 | 22.20640569 |
| P20959 | 1.89E+08 | 8.88E+06 | 21.28378378 |
| Q5NTB3 | 2.54E+08 | 1.47E+07 | 17.27891156 |
| Q29RQ1 | 1.51E+10 | 9.34E+08 | 16.16702355 |
| Q5E9E3 | 2.98E+09 | 2.07E+08 | 14.39613527 |
| Q2KIV9 | 4.10E+09 | 3.15E+08 | 13.01587302 |
| Q32LP0 | 1.87E+08 | 1.53E+07 | 12.22222222 |
| Q29RU4 | 2.15E+10 | 1.98E+09 | 10.85858586 |
| P50448 | 2.55E+10 | 2.41E+09 | 10.58091286 |
| P06868 | 7.41E+09 | 8.00E+08 | 9.2625 |
| P04815 | 1.22E+09 | 1.36E+08 | 8.970588235 |
| Q2TBI0 | 6.40E+09 | 8.63E+08 | 7.41599073 |
| Q28065 | 4.01E+10 | 6.88E+09 | 5.828488372 |
| P02777 | 1.05E+09 | 1.96E+08 | 5.357142857 |
| P82943 | 1.89E+08 | 3.71E+07 | 5.094339623 |
| P12799 | 7.02E+07 | 1.38E+07 | 5.086956522 |
| Q0VCX1 | 8.25E+09 | 1.71E+09 | 4.824561404 |
| Q2KJD0 | 3.77E+07 | 8.45E+06 | 4.461538462 |
| Q9TT36 | 4.43E+08 | 1.05E+08 | 4.219047619 |
| P81187 | 1.22E+10 | 3.45E+09 | 3.536231884 |
| P33433 | 1.67E+10 | 5.02E+09 | 3.326693227 |
| Q2KJF1 | 7.38E+09 | 2.40E+09 | 3.075 |
| P12763 | 4.15E+10 | 1.65E+10 | 2.515151515 |
| Q3SZ57 | 4.36E+09 | 1.97E+09 | 2.21319797 |
| Q5E9Z2 | 4.05E+09 | 1.92E+09 | 2.109375 |
| O02659 | 1.78E+09 | 9.57E+08 | 1.859979101 |
| P34955 | 3.40E+10 | 1.97E+10 | 1.725888325 |
| Q28178 | 4.83E+09 | 2.96E+09 | 1.631756757 |
| P02676 | 2.48E+08 | 1.68E+08 | 1.476190476 |
| Q3SZP2 | 3.78E+07 | 2.73E+07 | 1.384615385 |
| P12260 | 3.00E+08 | 2.27E+08 | 1.321585903 |
| Q3T052 | 2.23E+10 | 1.81E+10 | 1.232044199 |
| P23805 | 1.80E+10 | 1.57E+10 | 1.146496815 |
| Q17QH6 | 1.18E+08 | 1.04E+08 | 1.134615385 |
| Q3SZV7 | 1.97E+10 | 1.75E+10 | 1.125714286 |
| Q58D62 | 2.10E+09 | 1.88E+09 | 1.117021277 |
| P18902 | 2.95E+08 | 2.65E+08 | 1.113207547 |
| Q3MHN2 | 5.22E+09 | 4.85E+09 | 1.07628866 |
| Q3SX14 | 6.41E+09 | 6.25E+09 | 1.0256 |
| Q28107 | 1.09E+10 | 1.08E+10 | 1.009259259 |
| Q0VCM4 | 3.99E+06 | 4.00E+06 | 0.9975 |
| Q3MHN5 | 1.83E+10 | 1.98E+10 | 0.924242424 |
| Q2TBU0 | 2.08E+09 | 2.26E+09 | 0.920353982 |
| Q9TTE1 | 1.05E+10 | 1.21E+10 | 0.867768595 |
| Q58CQ9 | 1.96E+08 | 2.30E+08 | 0.852173913 |
| Q5E9F7 | 3.22E+07 | 3.78E+07 | 0.851851852 |
| Q2KJ83 | 1.45E+09 | 1.71E+09 | 0.847953216 |
| Q29443 | 2.57E+09 | 3.17E+09 | 0.810725552 |
| P38657 | 2.71E+07 | 4.29E+07 | 0.631701632 |
| P01966 | 9.63E+09 | 1.60E+10 | 0.601875 |
| Q5E9B1 | 1.10E+08 | 1.84E+08 | 0.597826087 |
| P02769 | 5.30E+11 | 8.87E+11 | 0.597519729 |
| Q03247 | 6.55E+08 | 1.12E+09 | 0.584821429 |
| P01030 | 2.77E+10 | 4.77E+10 | 0.580712788 |
| Q7SIH1 | 3.00E+09 | 5.22E+09 | 0.574712644 |
| P07589 | 8.83E+09 | 1.55E+10 | 0.569677419 |
| P10096 | 1.71E+08 | 3.24E+08 | 0.527777778 |
| P17697 | 6.38E+09 | 1.25E+10 | 0.5104 |
| Q6URK6 | 5.02E+06 | 9.91E+06 | 0.506559031 |
| O46375 | 5.82E+08 | 1.16E+09 | 0.501724138 |
| P84080 | 5.98E+06 | 1.26E+07 | 0.474603175 |
| Q5E9I6 | 5.98E+06 | 1.26E+07 | 0.474603175 |
| P01888 | 1.14E+08 | 2.81E+08 | 0.40569395 |
| Q0VCM5 | 5.17E+10 | 1.32E+11 | 0.391666667 |
| O77588 | 8.19E+07 | 2.29E+08 | 0.357641921 |
| P02672 | 1.56E+09 | 4.63E+09 | 0.336933045 |
| Q6R8F2 | 5.80E+07 | 1.74E+08 | 0.333333333 |
| P22226 | 1.17E+08 | 3.52E+08 | 0.332386364 |
| P28800 | 1.55E+09 | 5.10E+09 | 0.303921569 |
| Q2UVX4 | 1.28E+11 | 4.69E+11 | 0.272921109 |
| P02081 | 1.91E+09 | 7.24E+09 | 0.263812155 |
| P81947 | 1.15E+07 | 5.26E+07 | 0.218631179 |
| Q95M17 | 5.59E+07 | 2.57E+08 | 0.217509728 |
| P02639 | 9.39E+06 | 4.47E+07 | 0.210067114 |
| Q27970 | 8.96E+06 | 4.37E+07 | 0.205034325 |
| P00978 | 7.68E+09 | 3.81E+10 | 0.201574803 |
| Q32KY0 | 4.82E+08 | 2.45E+09 | 0.196734694 |
| P61223 | 2.81E+07 | 1.64E+08 | 0.171341463 |
| P15497 | 6.76E+10 | 3.99E+11 | 0.169423559 |
| Q3SWW8 | 3.23E+08 | 2.02E+09 | 0.15990099 |
| P01044 | 4.96E+09 | 3.15E+10 | 0.157460317 |
| A7E3W2 | 1.88E+07 | 1.26E+08 | 0.149206349 |
| P25326 | 4.07E+07 | 2.91E+08 | 0.139862543 |
| Q32PJ2 | 2.70E+09 | 1.99E+10 | 0.135678392 |
| P63103 | 1.22E+08 | 9.40E+08 | 0.129787234 |
| Q29437 | 1.99E+09 | 1.59E+10 | 0.125157233 |
| P81644 | 3.56E+09 | 3.01E+10 | 0.118272425 |
| P00735 | 1.04E+10 | 9.04E+10 | 0.115044248 |
| P80109 | 6.36E+08 | 5.57E+09 | 0.114183124 |
| P19858 | 6.72E+06 | 5.90E+07 | 0.113898305 |
| P01045 | 1.75E+09 | 1.65E+10 | 0.106060606 |
| P07224 | 8.92E+08 | 9.15E+09 | 0.097486339 |
| P19035 | 3.49E+09 | 4.02E+10 | 0.08681592 |
| P00745 | 3.87E+08 | 4.51E+09 | 0.085809313 |
| P37141 | 3.65E+07 | 5.49E+08 | 0.066484517 |
| Q3SZR3 | 2.54E+09 | 3.89E+10 | 0.06529563 |
| P00744 | 1.10E+08 | 1.93E+09 | 0.056994819 |
| P35445 | 1.95E+08 | 3.59E+09 | 0.054317549 |
| P35541 | 2.31E+08 | 4.35E+09 | 0.053103448 |
| P19034 | 1.92E+07 | 4.14E+08 | 0.046376812 |
| Q32L76 | 2.58E+08 | 5.93E+09 | 0.043507589 |
| P42916 | 6.01E+07 | 1.45E+09 | 0.041448276 |
| Q9BGI3 | 3.99E+07 | 9.76E+08 | 0.040881148 |
| Q3Y5Z3 | 8.77E+08 | 2.45E+10 | 0.035795918 |
| P00743 | 1.10E+08 | 3.26E+09 | 0.033742331 |
| P00741 | 3.34E+08 | 1.23E+10 | 0.027154472 |
| Q05443 | 1.31E+08 | 5.14E+09 | 0.025486381 |
| P02453 | 9.47E+07 | 3.77E+09 | 0.025119363 |
| P80012 | 2.37E+08 | 9.63E+09 | 0.024610592 |
| P62261 | 1.12E+07 | 6.36E+08 | 0.017610063 |
| P56652 | 5.20E+08 | 3.03E+10 | 0.017161716 |
| Q0VCX2 | 5.22E+06 | 3.07E+08 | 0.017003257 |
| P22457 | 4.30E+06 | 6.09E+08 | 0.007060755 |
| O77742 | 1.26E+07 | 2.77E+09 | 0.004548736 |

| **Table S2. RT-qPCR primer sequences.** | |
| --- | --- |
| **Gene name RT-qPCR primer sequence (5’-3’)** | |
| *Gapdh-*F | TGGCCTTCCGTGTTCCTAC |
| *Gapdh-*R | GAGTTGCTGTTGAAGTCGCA |
| *Hmgcr-*F | AGCTTGCCCGAATTGTATGTG |
| *Hmgcr-*R | TCTGTTGTGAACCATGTGACTTC |
| *Fdps-*F | TCCAGGTCCAGGACGACTACCTTG |
| *Fdps-*R | CCCATAATTCTCCTCTAAGATCTGG |
| *Fdft1-* F | ATGGAGTTCGTCAAGTGTCTAGG |
| *Fdft1-*R | CGTGCCGTATGTCCCCATC |
| *Sqle-*F | ATAAGAAATGCGGGGATGTCAC |
| *Sqle-*R | ATATCCGAGAAGGCAGCGAAC |
| *Acat2-*F | CCCGTGGTCATCGTCTCAG |
| *Acat2-*R | GGACAGGGCACCATTGAAGG |
| *Scd1-*F | GCAAGCTCTACACCTGCCTCTT |
| *Scd1-*R | CGTGCCTTGTAAGTTCTGTGGC |
| *Acsl3-*F | GGGACTACAATACCGGCAGA |
| *Acsl3-*R | ATAGCCACCTTCCTCCCAGT |
| *Elov6-*F | CGGCATCTGATGAACAAGCGAG |
| *Elov6-*R | GTACAGCATGTAAGCACCAGTTC |
| *Hmgcs1-*F | GCCGTGAACTGGGTCGAA |
| *Hmgcs1-*R | GCATATATAGCAATGTCTCCTGCAA |
| *Mvk-*F | AGGTCCCGCGGAGTACCAAG |
| *Mvk-*R | CTAGCACGCGCTCACACTCC |
| *Pmvk-*F | AGGAGTATGCTCGGGAGCATG |
| *Pmvk-*R | TGTGTCACTCACCAGCCAGATAG |
| *Mvd-*F | CAGCCAATGGAGACAAGTTCC |
| *Mvd-*R | GTCCTGGTCCGACCTGAGTG |
| *Cyp51a1-*F | ACTTACGACCAGTTGAAGGATCTG |
| *Cyp51a1-*R | CCTGCCACCGTCTGAGGGGTC |
| *Lbr-*F | ATGCCAAGTAGGAAGTTTGTTGA |
| *Lbr-*R | GATTTGTTGTCGTGGCTCAGA |
| *Msmo1-*F | ACGAGTTTCAGGCTCCATTTGG |
| *Msmo1-R* | CATAACCGCTGTGCACATCG |
| *Nsdhl-*F | CAGGAGAGAGCAGTACTGGATG |
| *Nsdhl-*R | CAGGTTTTCCCCATTTCCAATC |
| *Hsd17b7-*F | CTGTGACACCGTACAACGGA |
| *Hsd17b7-*R | GCTCGGGTGATCCGATTTCT |
| *Dhcr24-*F | CTCTGGGTGCGAGTGAAGG |
| *Dhcr24-*R | TTCCCGGACCTGTTTCTGGAT |
| *Ebp-*F | ATGACCACCAATACGGTCCC |
| *Ebp-*R | GCCAACCAGGATATGCGAAGT |
| *Dhcr7-*F | GGGCTGCAAGCCTGGCTCATT |
| *Dhcr7-*R | TGCGAACGTGGACACGGCAT |
| *Srebp1-*F | GGCCGAGATGTGCGAACT |
| *Srebp1-*R | TTGTTGATGAGCTGGAGCATGT |
| *Srebp2-*F | GCGTTCTGGAGACCATGGA |
| *Srebp2-*R | ACAAAGTTGCTCTGAAAACAAATCA |
| *Sc5d-*F | ACTTTCCAAATGGCTGGATTCATC |
| *Sc5d-*R | TATGTATGCGCTTGTAGACCAG |
| *Acsl4-*F | TCCTCCAAGTAGACCAACCCC |
| *Acsl4-*R | AGTCCAGGGATACGTTCACAC |
| *Fads1-*F | CACCCTTTGGATCTTTGGAA |
| *Fads1-*R | GCATGGGGATATGGTTCATC |
| *Fads2-*F | TTCCTGGAGAGCCACTGGTTTG |
| *Fads2-*R | GAAGAAGGACTGCTCCACATTGC |
| *Scd2-*F | AGATGATCTATATGACCCCACCT |
| *Scd2-*R | CCCAGGGCGCTGATTACATA |
