# Supplementary material for: Serum apolipoprotein H determines ferroptosis resistance by modulating cellular lipid composition
Source: Cell Death Dis. 2024 Oct 1;15(10):718. doi: 10.1038/s41419-024-07099-2 (PMC11445452; doi:10.1038/s41419-024-07099-2)

Unprocessed images for Fig. 3E

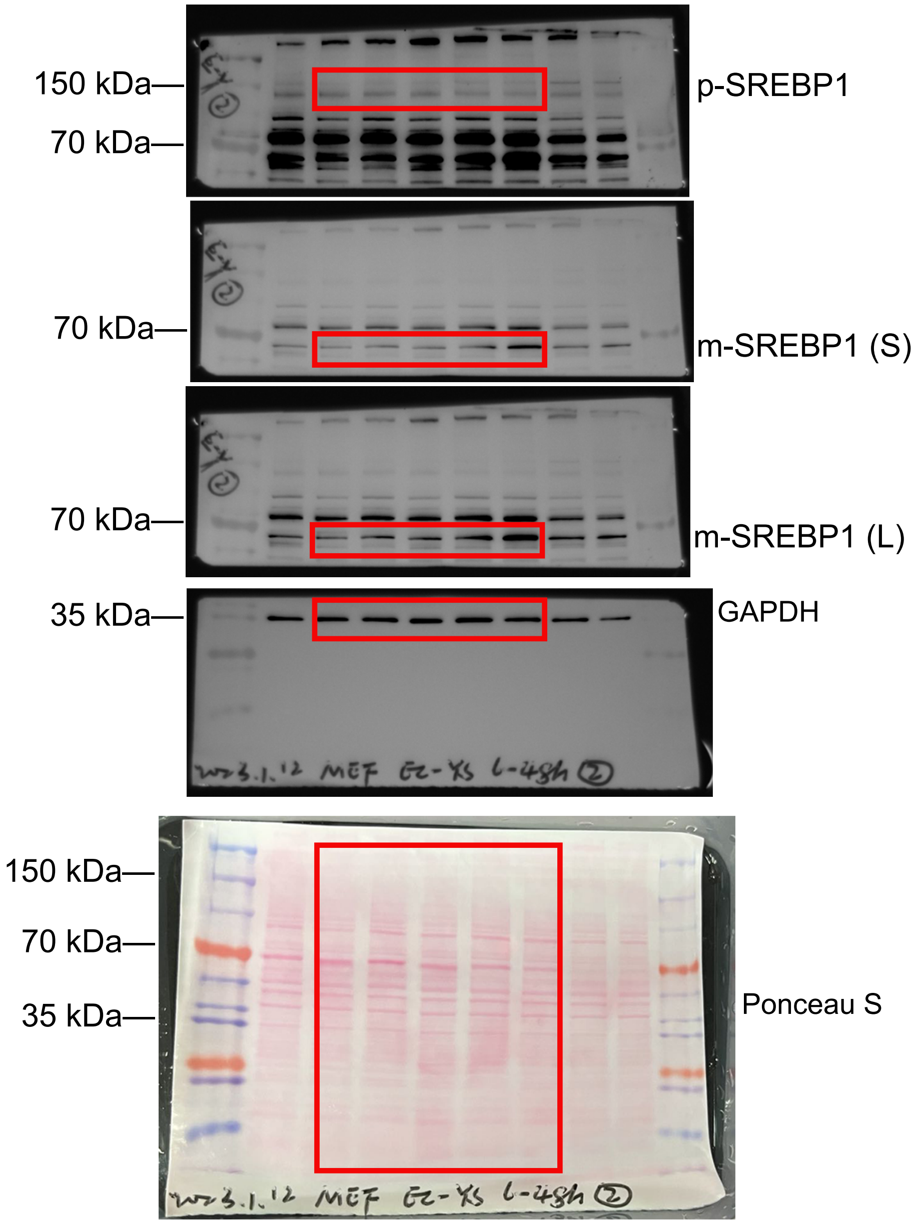

Unprocessed images for Fig. 4D

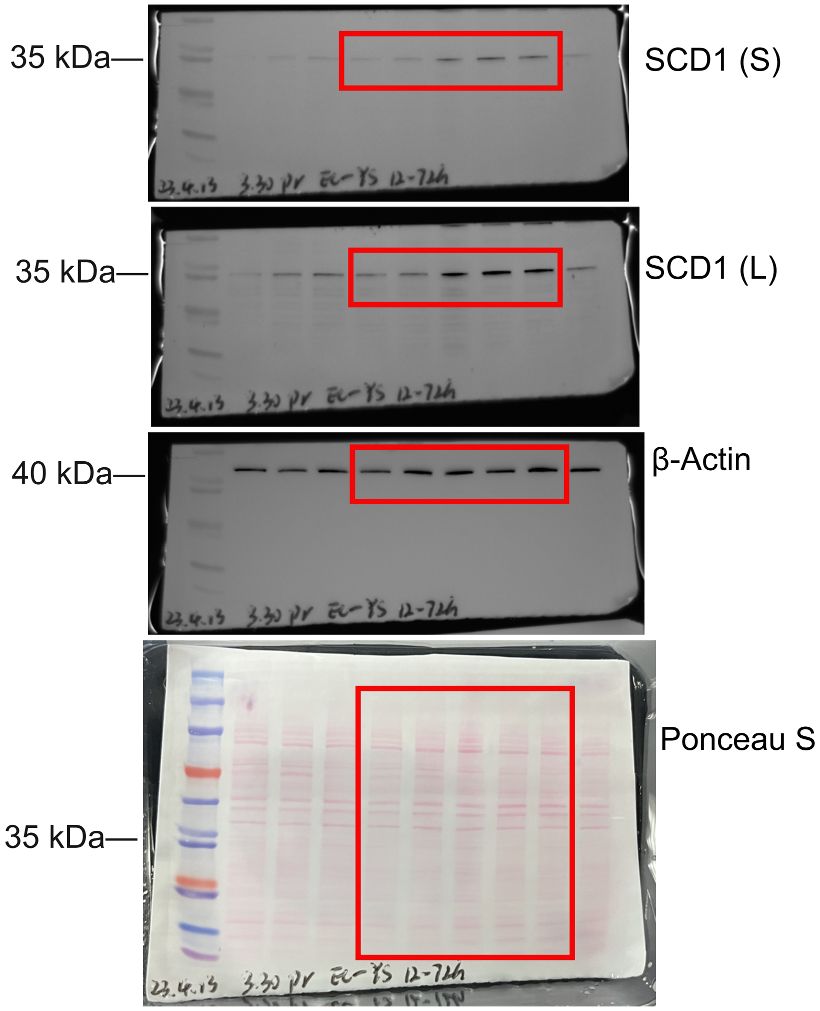

Unprocessed images for Fig. 4E

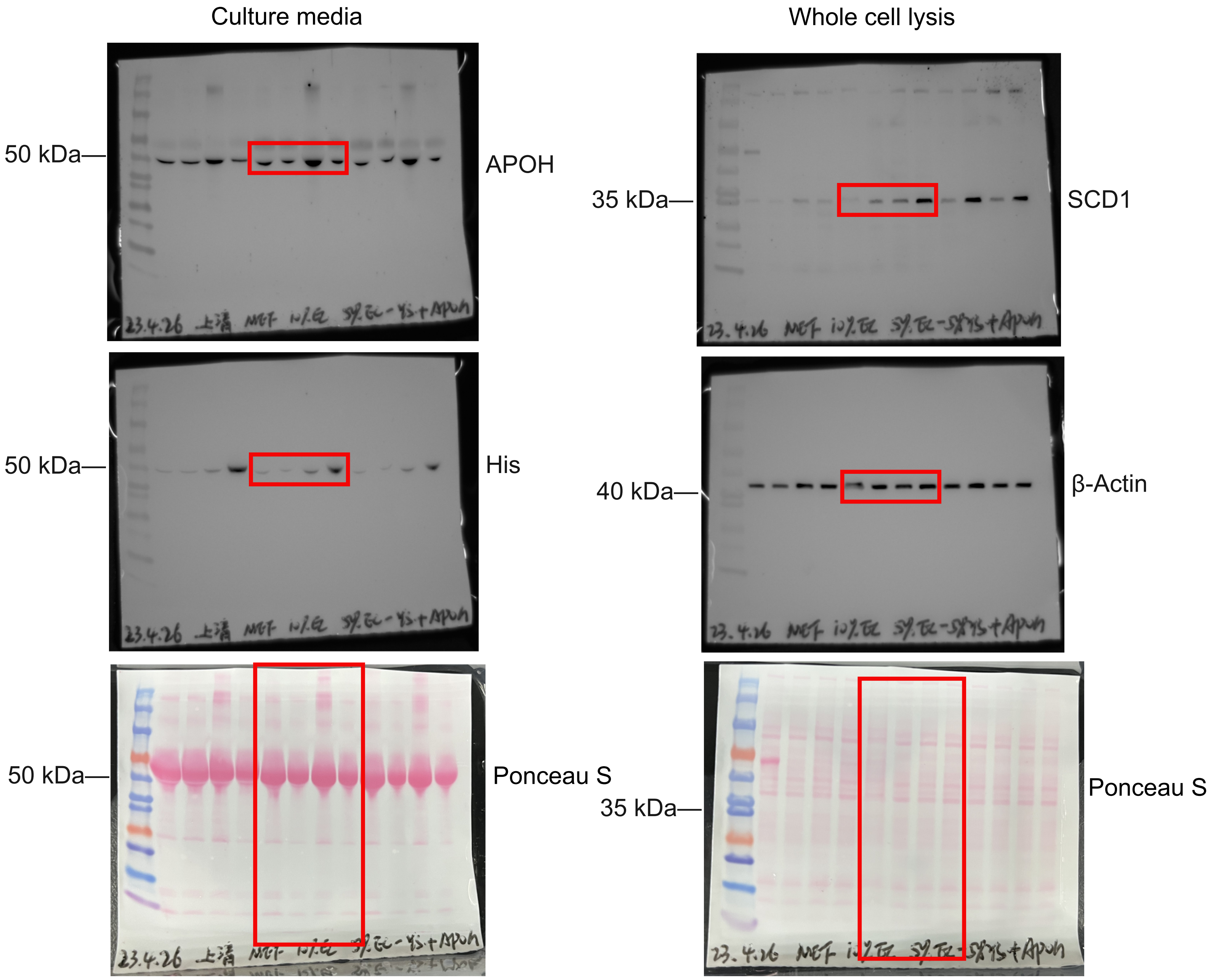

Unprocessed images for Fig. 5A

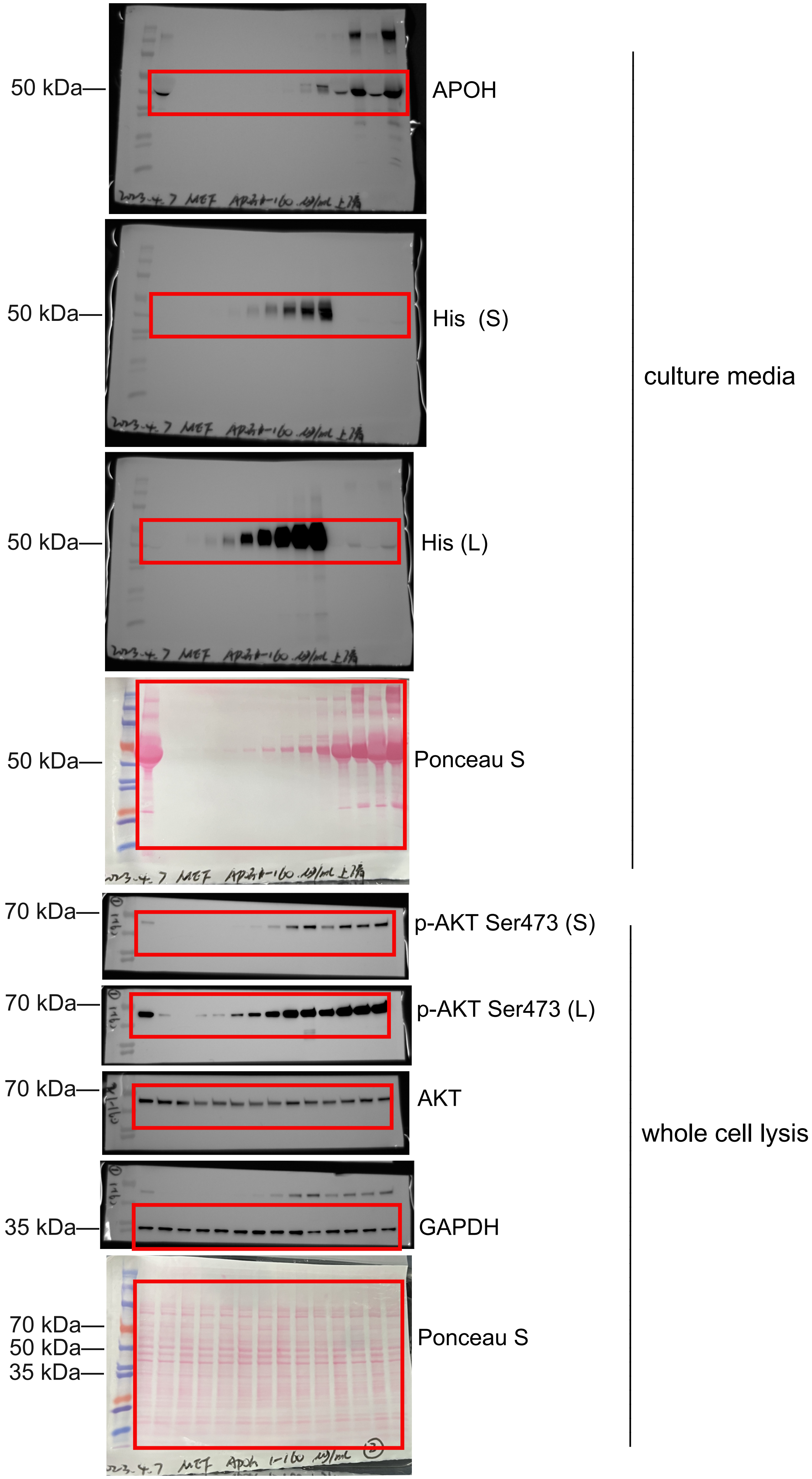

Unprocessed images for Fig. 5B

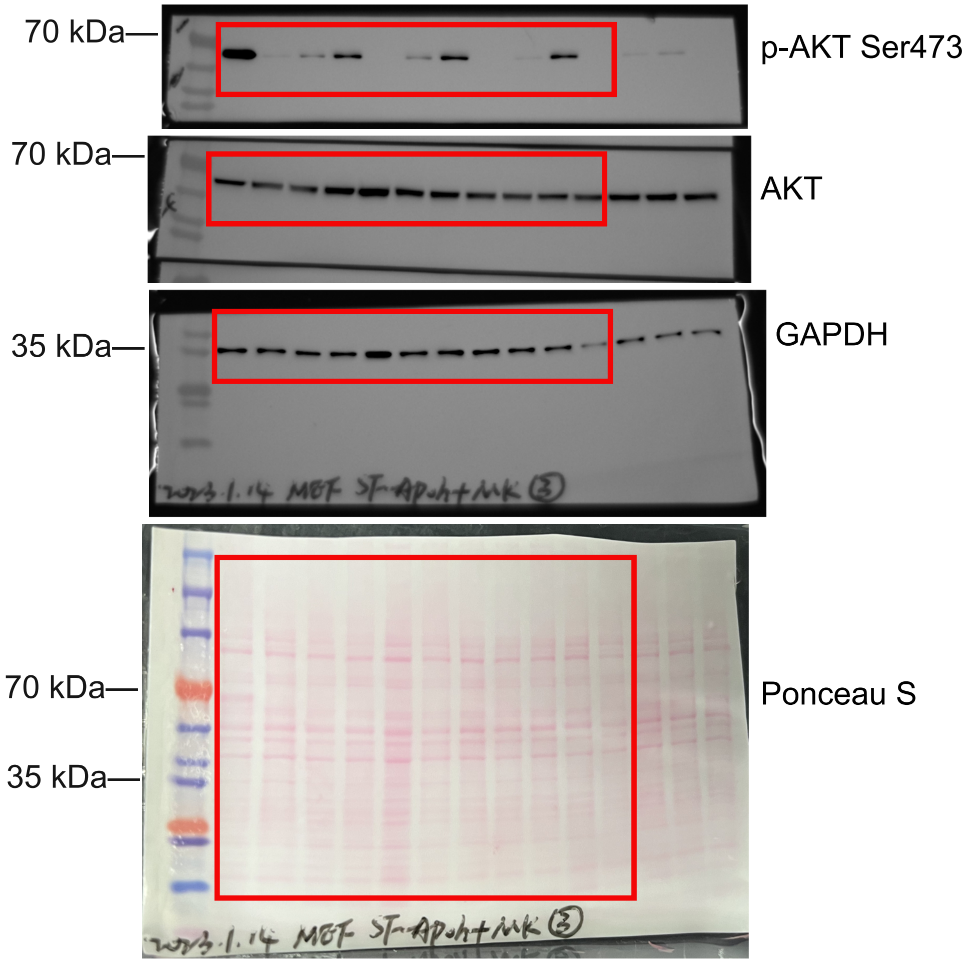

Unprocessed images for Fig. S2B

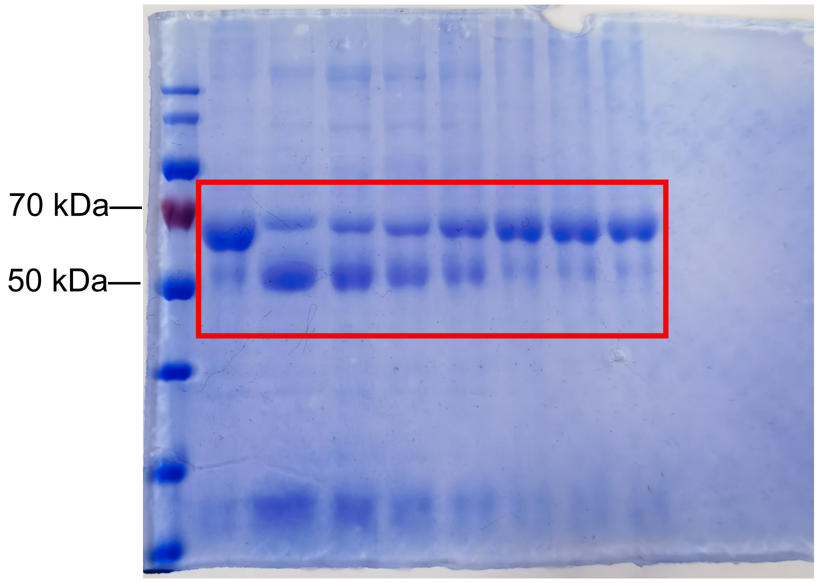

Unprocessed images for Fig. S2F

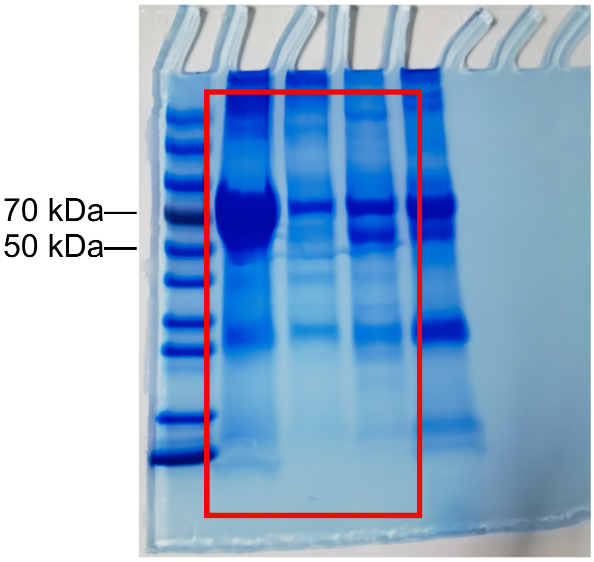

Unprocessed images for Fig. S2G

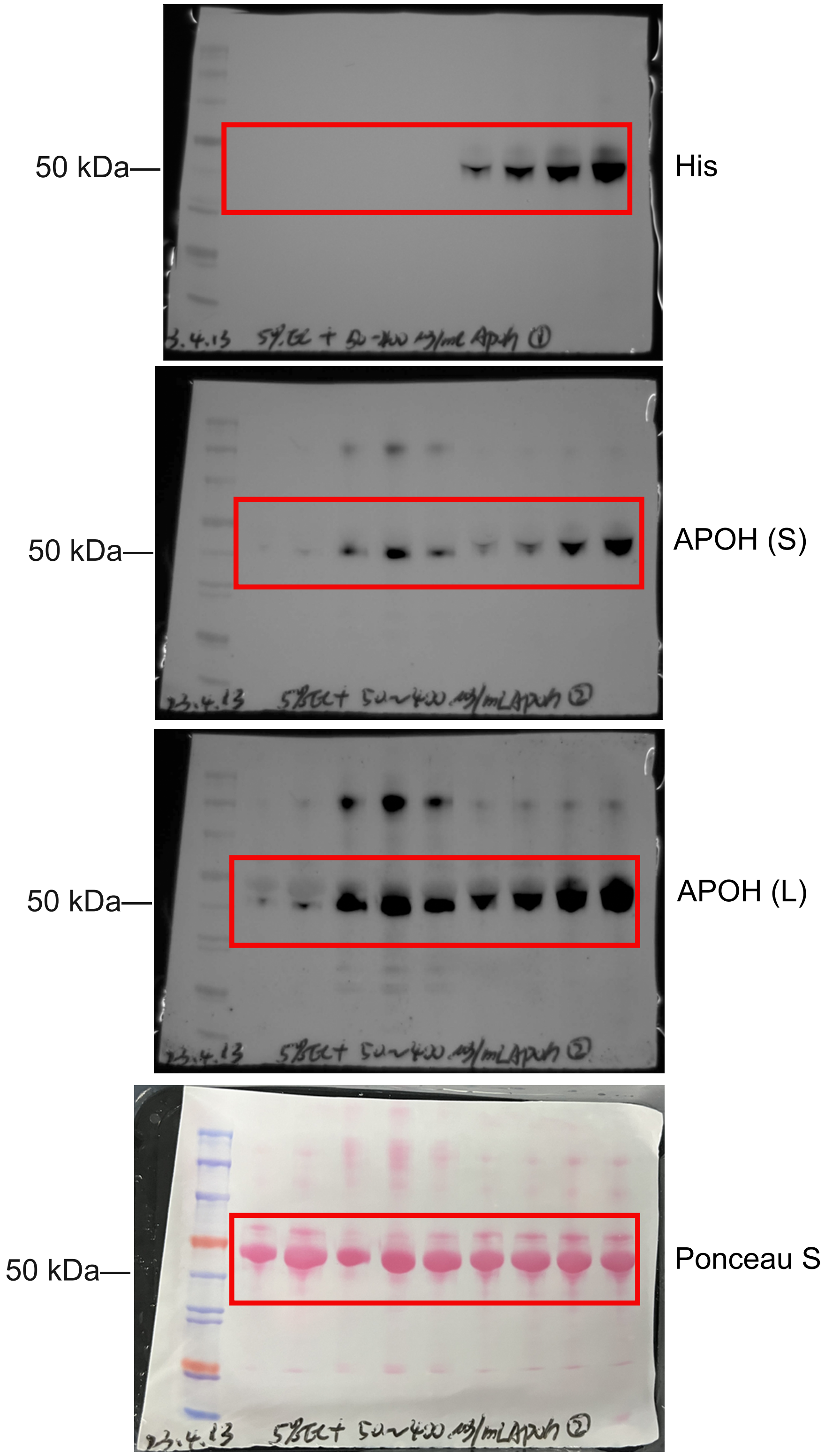

Unprocessed images for Fig. S2I

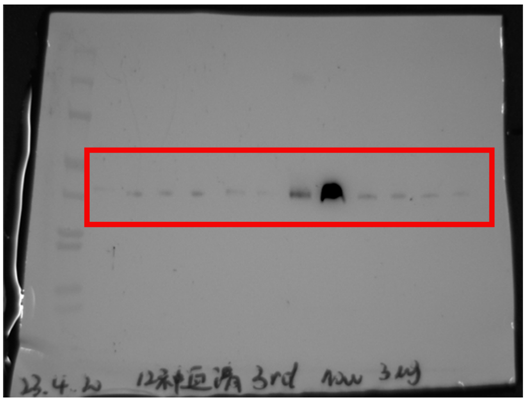

50 kDa— APOH (S)

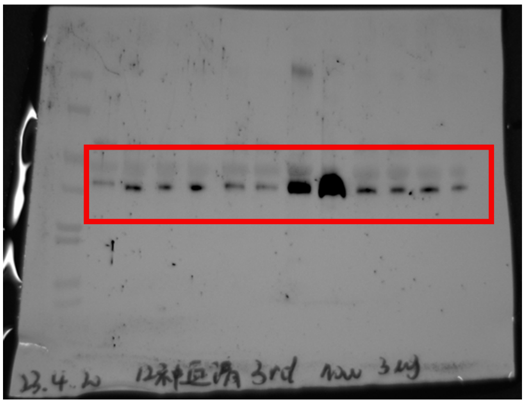

50 kDa— APOH (L)

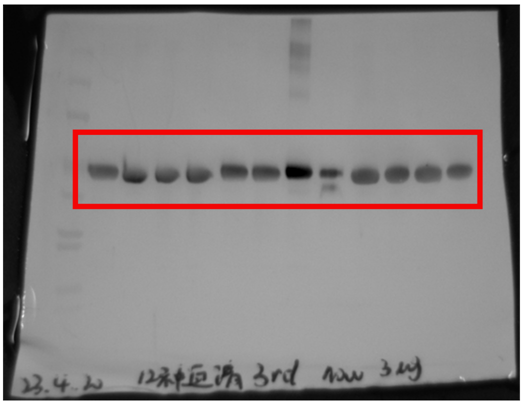

50 kDa— Albumin

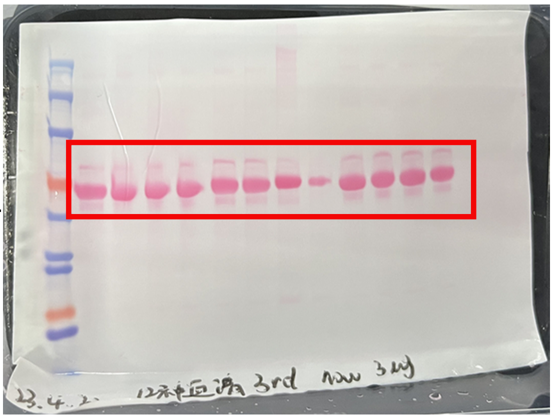

50 kDa— Ponceau S

Unprocessed images for Fig. S2J

50 kDa—

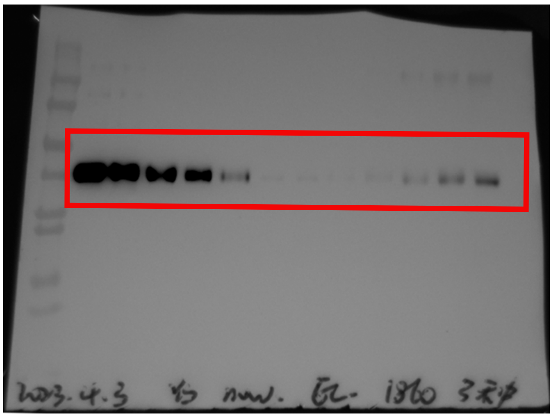

APOH (S)

50 kDa—

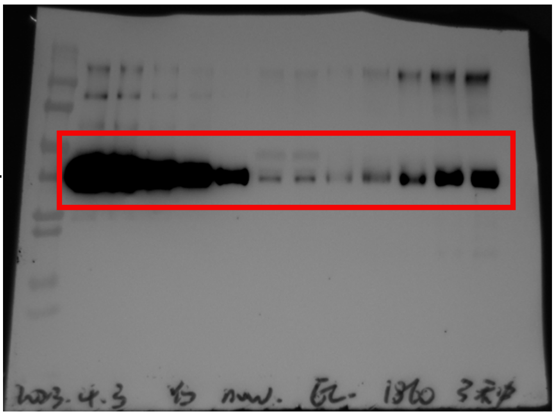

APOH (L)

50 kDa—

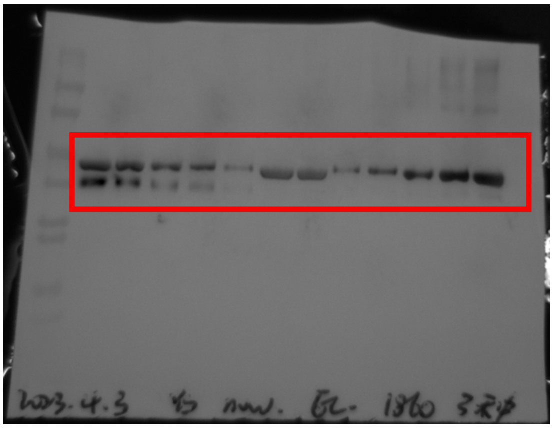

Albumin

50 kDa—

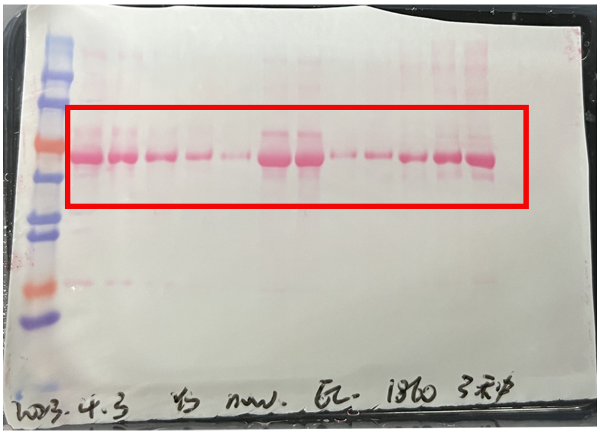

Ponceau S

Unprocessed images for Fig. S5A

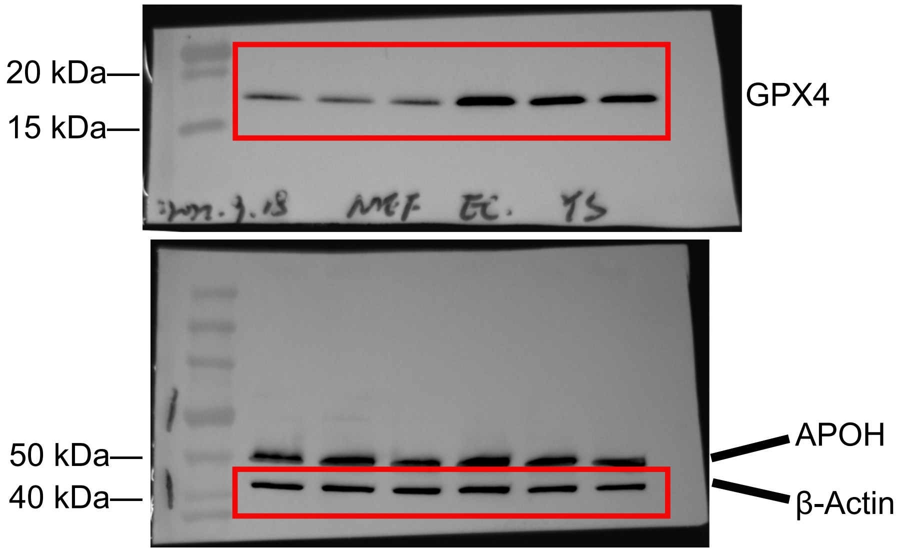

Unprocessed images for Fig. S5B

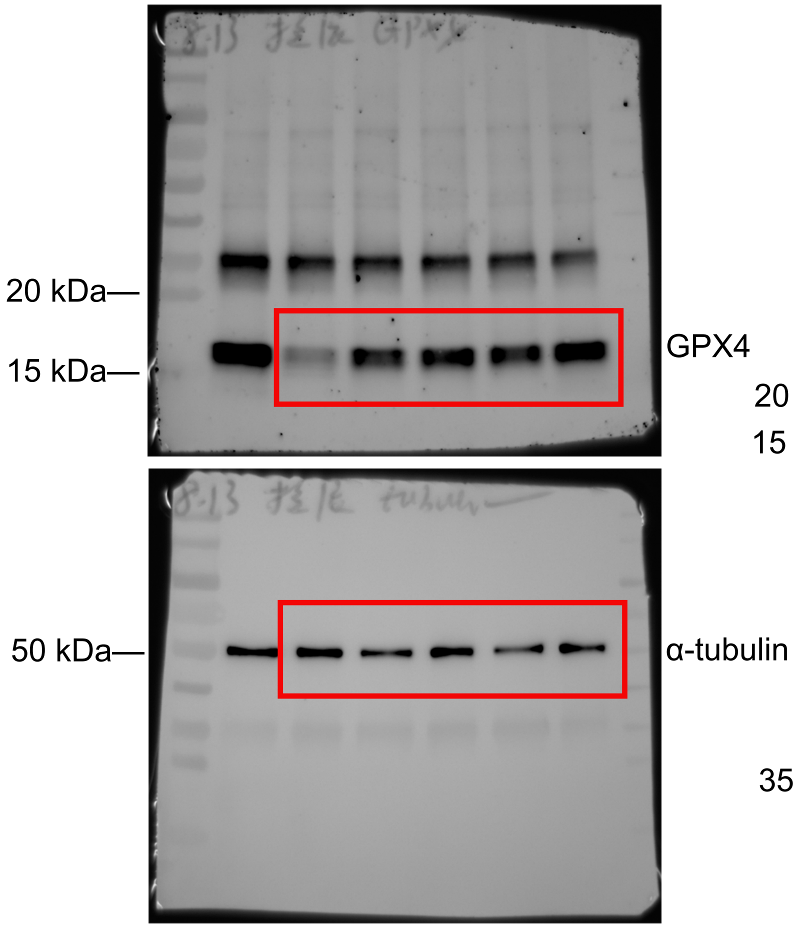

Unprocessed images for Fig. S5E

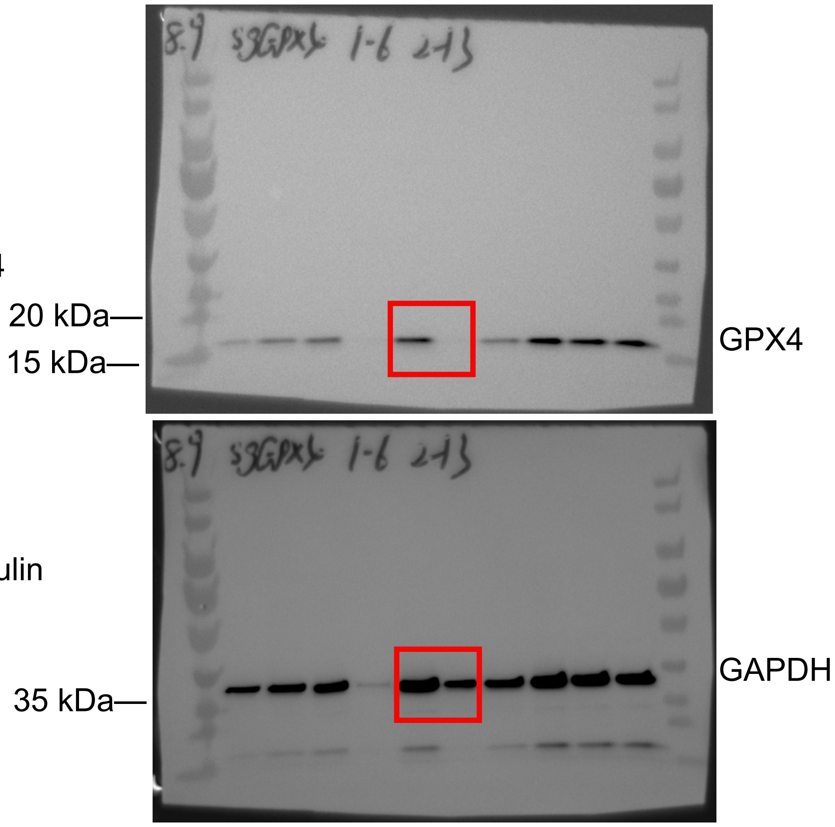

Unprocessed images for Fig. S6A

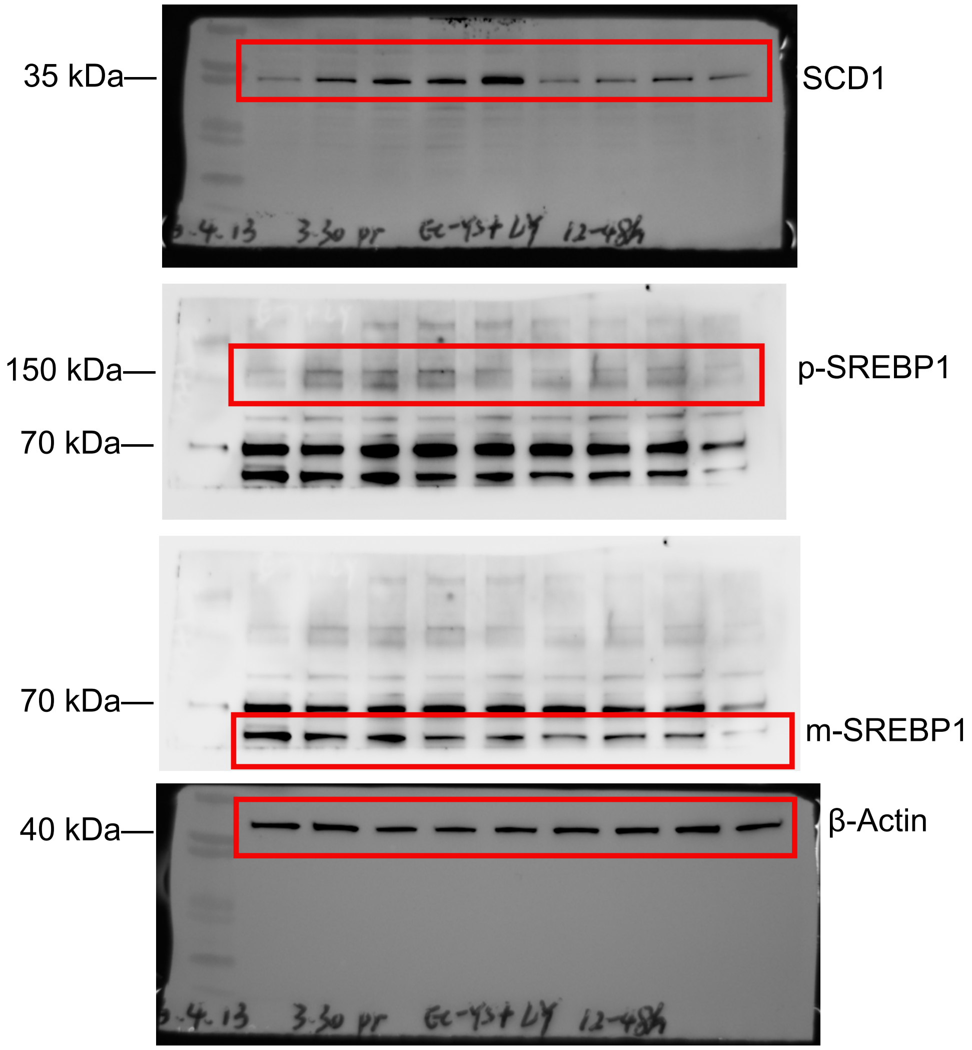

Unprocessed images for Fig. S6B

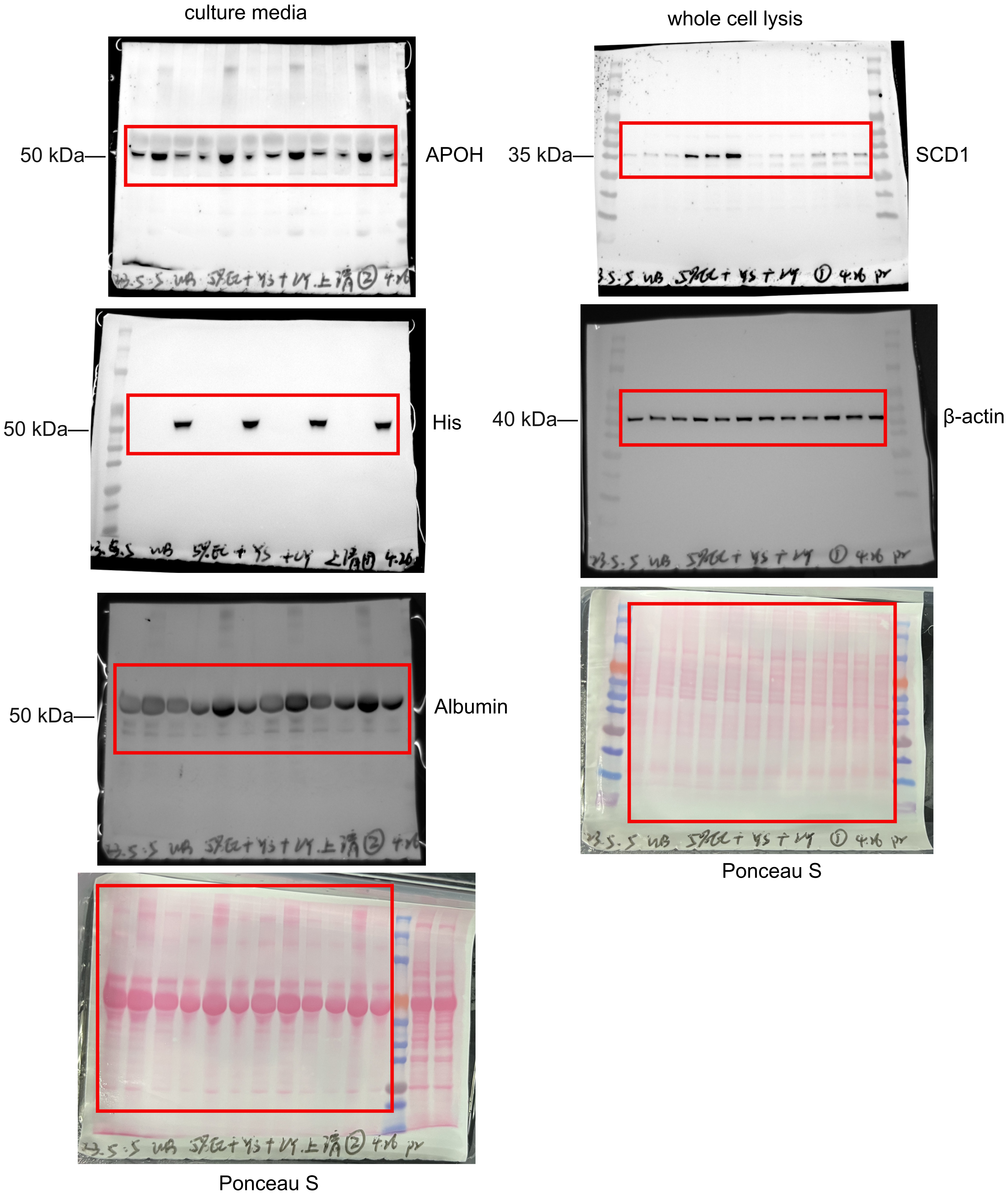

Unprocessed images for Fig. S6C

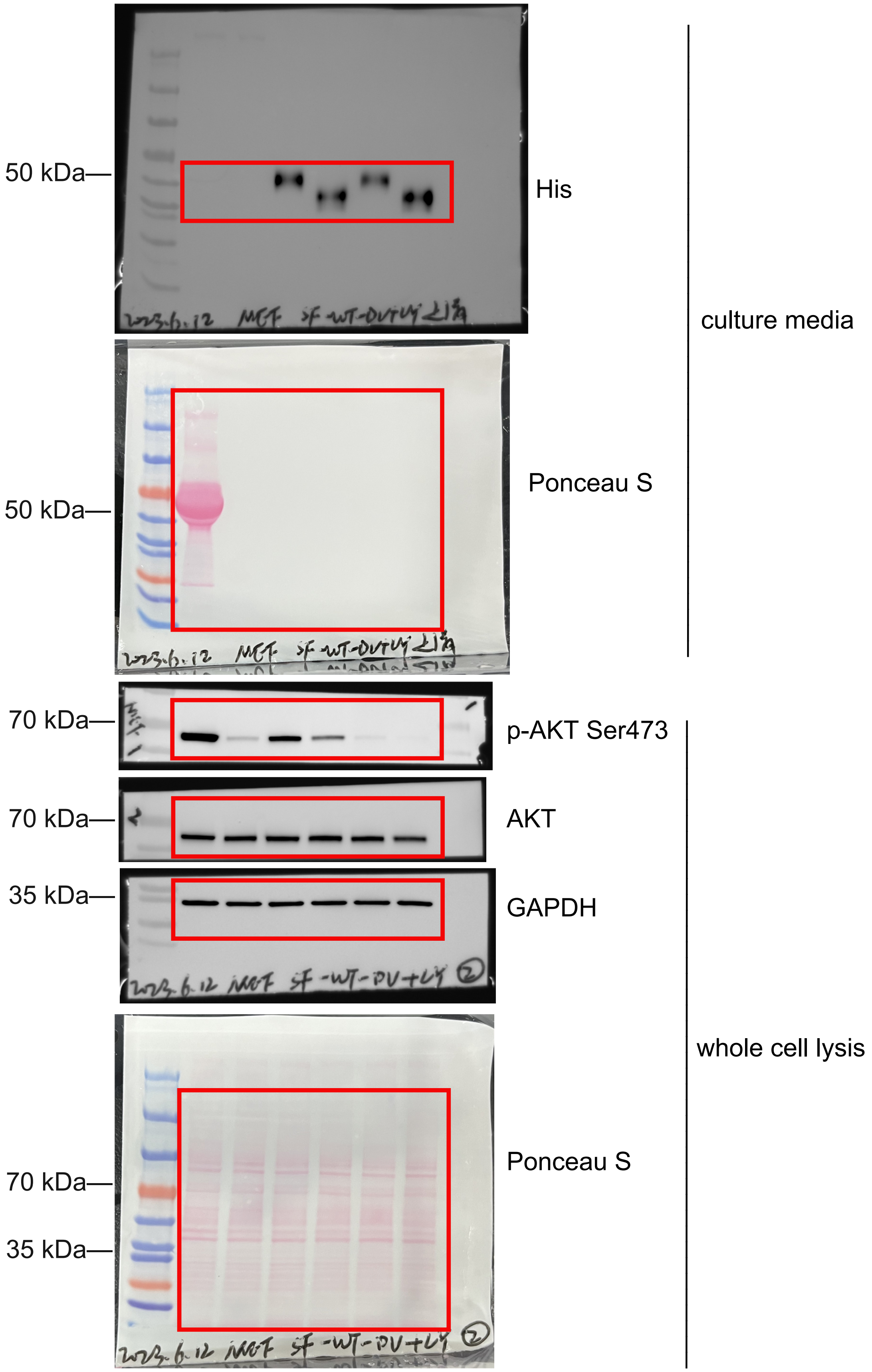

Unprocessed images for Fig. S6D

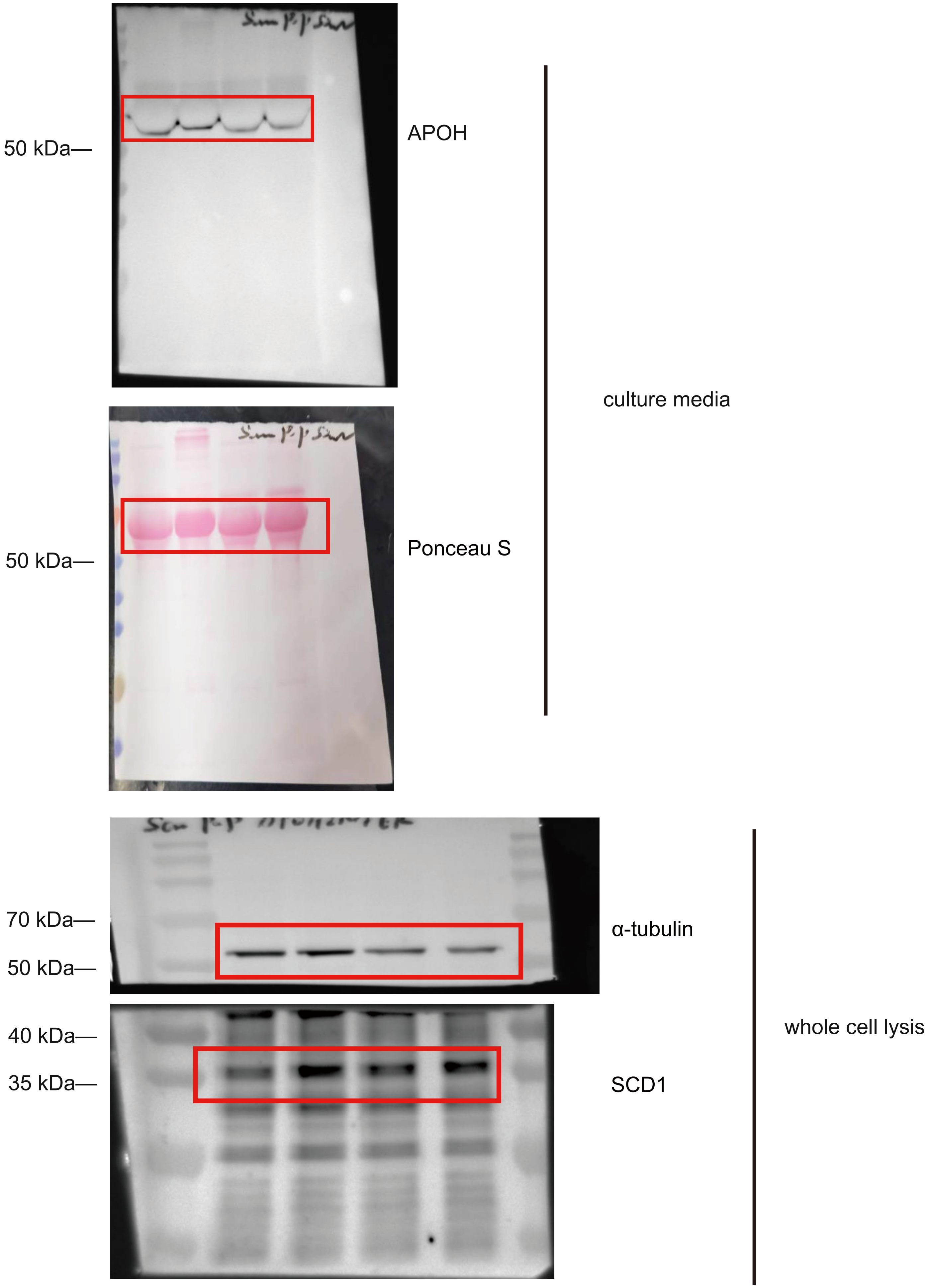

Supplement: Supplementary file 2 — Original Western Blot [file 41419_2024_7099_MOESM2_ESM.pdf]
